# Identifying HLA *DRB1-DQB1* alleles associated with *Chlamydia trachomatis* infection and in silico prediction of potentially-related peptides

**DOI:** 10.1038/s41598-021-92294-w

**Published:** 2021-06-18

**Authors:** Leidy Pedraza, Milena Camargo, Darwin A. Moreno-Pérez, Ricardo Sánchez, Luisa Del Río-Ospina, Indira M. Báez-Murcia, Manuel E. Patarroyo, Manuel A. Patarroyo

**Affiliations:** 1 Molecular Biology and Immunology Department, Fundación Instituto de Inmunología de Colombia (FIDIC), 111321 Bogotá D.C., Colombia; 2 MSc Programme in Microbiology, Universidad Nacional de Colombia, 111321 Bogotá D.C., Colombia; 3 Animal Science Faculty, Universidad de Ciencias Aplicadas y Ambientales (U.D.C.A), 111166 Bogotá D.C., Colombia; 4 Faculty of Medicine, Universidad Nacional de Colombia, 111321 Bogotá D.C., Colombia; 5 Health Sciences Division, Main Campus, Universidad Santo Tomás, 110231 Bogotá D.C., Colombia

**Keywords:** Immunogenetics, Bacterial infection

## Abstract

HLA class II (HLA-II) genes’ polymorphism influences the immune response to *Chlamydia trachomatis* (*Ct*), it is considered a sexually transmitted infection. However, associations between HLA-II alleles and *Ct*-infection have been little explored in humans; this study was thus aimed at determining HLA-*DRB1*-*DQB1* alleles/haplotypes’ effect on *Ct*-infection outcome in a cohort of Colombian women. Cervical sample DNA was used as template for detecting *Ct* by PCR and typing HLA-*DRB1*-*DQB1* alleles/haplotypes by Illumina MiSeq sequencing. Survival models were adjusted for identifying the alleles/haplotypes’ effect on *Ct-*outcome; bioinformatics tools were used for predicting secreted bacterial protein T- and B-cell epitopes. Sixteen HLA-*DRB1* alleles having a significant effect on *Ct*-outcome were identified in the 262 women analysed. DRB1*08:02:01G and DRB1*12:01:01G were related to infection-promoting events. Only the DQB1*05:03:01G allele related to clearance/persistence events was found for HLA-*DQB1*. HLA-*DRB1* allele homozygous women were associated with events having a lower probability of clearance and/or early occurrence of persistence. Twenty-seven peptides predicted in silico were associated with protective immunity against *Ct*; outer membrane and polymorphic membrane protein-derived peptides had regions having dual potential for being T- or B-cell epitopes. This article describes HLA-*DRB1*-*DQB1* alleles/haplotypes related to *Ct*-infection resolution and the peptides predicted in silico which might probably be involved in host immune response. The data provides base information for developing future studies leading to the development of effective prevention measures against *Ct*-infection.

## Introduction

*Chlamydia trachomatis* (*Ct*) is the commonest bacteria-related, sexually-transmitted infection (STI) worldwide^1^; the WHO’s Report on global sexually transmitted infection surveillance estimated that there are 127 million new cases annually^2^. Most *Ct* infections have an asymptomatic clinical course, but some might lead to severe complications, such as pelvic inflammatory disease (PID) and recurrent abortions^1^.

*Ct* mainly affects 18–20 year-old women and 20–24 year-old men, asymptomatic cases mainly occurring in women (close to 90%)^3^; however, symptomatic infections usually occur weeks or months after exposure. Abundant secretion, dysuria and postcoital bleeding are amongst the commonest symptoms^3^.

Previous studies have revealed that late detection of *Ct* asymptomatic infection (together with other agents such HPV) could lead to conditions such as squamosas cell carcinoma and cervical cancer (CC)^4^. It has been shown that a fourth of *Ct* infections become spontaneously resolved and without treatment; factors, such as host immune response, thus significantly contribute towards eliminating genital tract infection^5^.

The major histocompatibility complex (MHC) is a mechanism greatly determining infections’ clinical course; establishing its role in *Ct* infection dynamics would thus explain their outcome^5^. Some studies have suggested the relationship between class II HLA alleles, *Ct* and tubal factor infertility (TFI)^6^ as DQA*03:01, DQA*05:01 and DQB*04:02 have been described as being associated with *Ct* and DRB1*75:03 and DRB5*01:01 with TFI^6^*.*

An efficient immune response against intracellular pathogens like *Ct* requires cell-mediated immunity, this being stimulated by bacterial peptides presented to T-cells by the MHC^7^. Predicting these molecules and establishing their potential role in the natural history of *Ct* infection could contribute towards understanding its dynamics^8^.

Studies have searched for prophylactic interventions; most have evaluated the immune response in mice experimentally infected by *Ct* or *C. muridarum*^9,10^. Murine model studies have demonstrated the importance of an HLA class II molecule-mediated immune response regarding bacterial infection resolution^11^. However, few studies have comprehensively evaluated HLA alleles’ role regarding human *Ct* infection outcome and the peptides probably related to immune response^10,12^. This study was thus aimed at determining HLA-*DRB1*-*DQB1* alleles/haplotypes’ effect on *Ct* infection, persistence, clearance and redetection in a cohort of Colombian women. The results provided information for developing suitable prevention measures for managing and controlling *Ct* infection.

## Results

### HLA-*DRB1-DQB1* allele effects on *Ct* outcome

**Figure 1:**
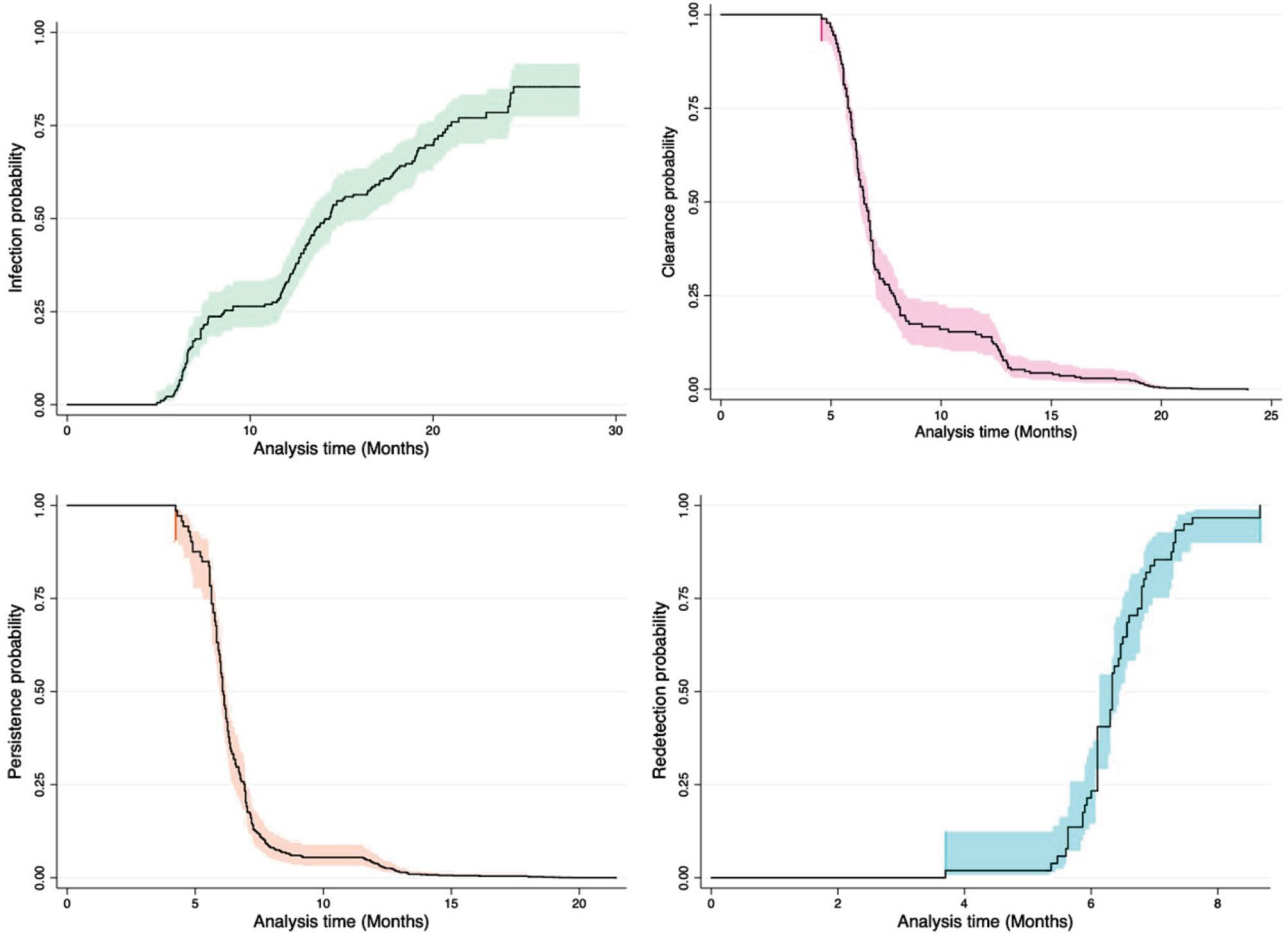
Kaplan–Meier estimator for evaluating Ct infection, clearance, persistence and redetection.

The demographic characteristics of 262 women complying with the retrospective study’s inclusion criteria were analysed; mean age was 41.7 years-old (23.1 SD) and median age at onset of sexual life 18.0 years-old (4.0 IQR) (Table 1). *Ct* was detected retrospectively; study results gave the highest rate for persistence events (26.0 per 100 women/month), followed by clearance (16.1 per 100 women/month) and redetection events (15.6 per 100 women/month) (Supplementary Fig. S1). Survival data was estimated (using Kaplan Meier survival functions) for each event; Fig. 1 shows the probability of Ct infection, clearance, persistence and redetection risk throughout the follow-up period.

**Table 1:** Base line demographic characteristics and risk factors for the 262 women included in the study.

Variable	Years	
Mean age (SD)	41.7 (23.1)	
Median age at first intercourse (IQR)	18.0 (4.0)	
Years of active sex life (SD)	23.1 (10.6)	

	*n*	(%)
Ethnicity—not mestizo^a^	7	(2.6)
More than three lifetime sexual partners	55	(21.8)
Marital status—married/cohabiting	231	(88.1)
Having had more than two pregnancies	37	(14.5)
A history of abortions	93	(35.5)
A history of sexually-transmitted diseases	60	(22.9)
Contraceptive method—hormonal	29	(11.9)
Educational level—illiterate/primary	122	(47.8)
Living outside Bogotá	180	(68.7)
Average monthly income—less than minimum wage^b^	232	(89.6)
HPV positive	218	(83.2)
Cytological findings—abnormal	23	(8.9)

^a^This category included Indigenous and Afro Colombian ethnicities.

^b^The Colombian minimum average monthly income would be roughly US$ 250.

Forty-seven HLA-*DRB1* alleles were found using Illumina MiSeq sequencer whose frequency distribution has been previously published^13^. The DRB1*04:07:01G allele had the greatest prevalence in the target population (Supplementary Table S1); this allele has been categorised as a common allele and has been reported in all continents’ populations^14^. Multivariate models (parametric and semiparametric) were constructed for identifying data associated with *Ct* outcomes; they were adjusted to fit the covariables associated with outcomes in univariate analysis (Supplementary Table S2).

Sixteen *DRB1* alleles had a statistically significant *p* value, indicating their possible effect regarding a particular infection event (greater probability (GP), earlier occurrence (EO), later occurrence (LO) and lower probability (LP) (Table 2 and Supplementary Tables S3 and S4). Interestingly, three alleles were related to more than one event; DRB1*08:02:01G and DRB1*12:01:01G had concordant effects (GP for persistence and EO for redetection and LO for infection and GP for clearance) while DRB1*14:02:01G had an opposite effect (LO for infection and GP for persistence) (Table 2). Fourteen HLA-*DQB1* alleles were identified in the target population (Supplementary Table S1), of which only DQB1*05:03:01G had a statistically significant *p* value related to clearance and persistence events, having a GP effect for both (Table 2 and Supplementary Tables S5 and S6). It was also found that HLA-*DRB1* homozygous women had a LP effect on clearance and an EO effect related to persistence events, suggesting that this characteristic could represent a genetic disadvantage for its carriers as it makes them more susceptible to *Ct* infection. However, more experimental evidence is required to confirm such hypothesis.

**Table 2:**
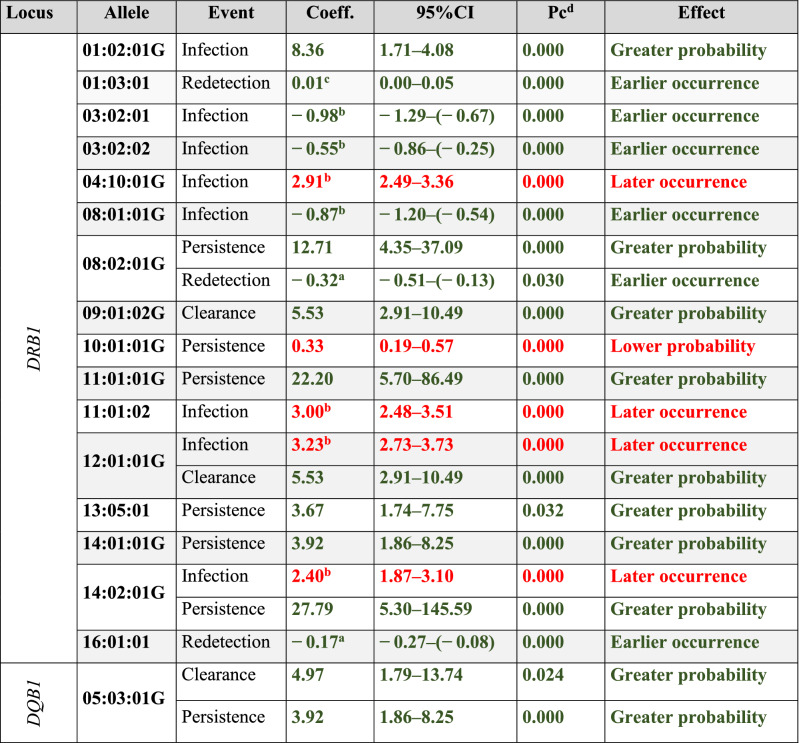
HLA *DRB1-DQB1* alleles associated with *Ct* infection, clearance, persistence and redetection.

Alleles affecting an event are shown as green associations [i.e. greater probability (GP) or earlier occurrence (EO)] and those hindering them [lower probability (LP) or later occurrence (LO)] as red associations.

*Pc* corrected *p* value, *95%CI* 95% confidence interval, *Coeff* regression coefficient, *DRB1* DR beta 1, *DQB1* DQ beta 1.

Values in bold indicate statistical significance based on 95%CI, *p* < 0.05.

^a^The Cox proportional hazards model did not fulfil the assumption of proportionality; a logistic parametric model was constructed.

^b^The Cox proportional hazards model did not fulfil the assumption of proportionality; a lognormal parametric model was constructed.

^c^The Cox proportional hazards model did not fulfil the assumption of proportionality; a Gompertz parametric model was constructed.

^d^The Bonferroni method was used for correcting all the models’ *p*-values.

Analysis was adjusted for age, onset of sexual life, lifetime amount of sexual partners, planning method, abortions, history of other STI and HPV infections.

### HLA-*DRB1*-*DQB1* haplotype effect on *Ct* outcome

Forty-seven of the 142 *DRB1-DQB1* haplotypes had statistically significant values when analysing the effect of each haplotype on *Ct* outcome (Table 3). Twenty-seven associations were found regarding infection events; 16 related to the event (GP/EO) (e.g. DRB1*01:02:01G-DQB1*03:03:02G) whilst 11 associations did not (LO) (e.g. DRB1*12:01:01G-DQB1*03:02:01G). DRB1*09:01:02G-DQB1*03:01:01G and DRB1*12:01:01G-DQB1*03:02:01G had an EO effect on clearance whilst DRB1*04:05:01-DQB1*03:01:01G was associated with LP (Table 3 and Supplementary Table S7).

**Table 3:**
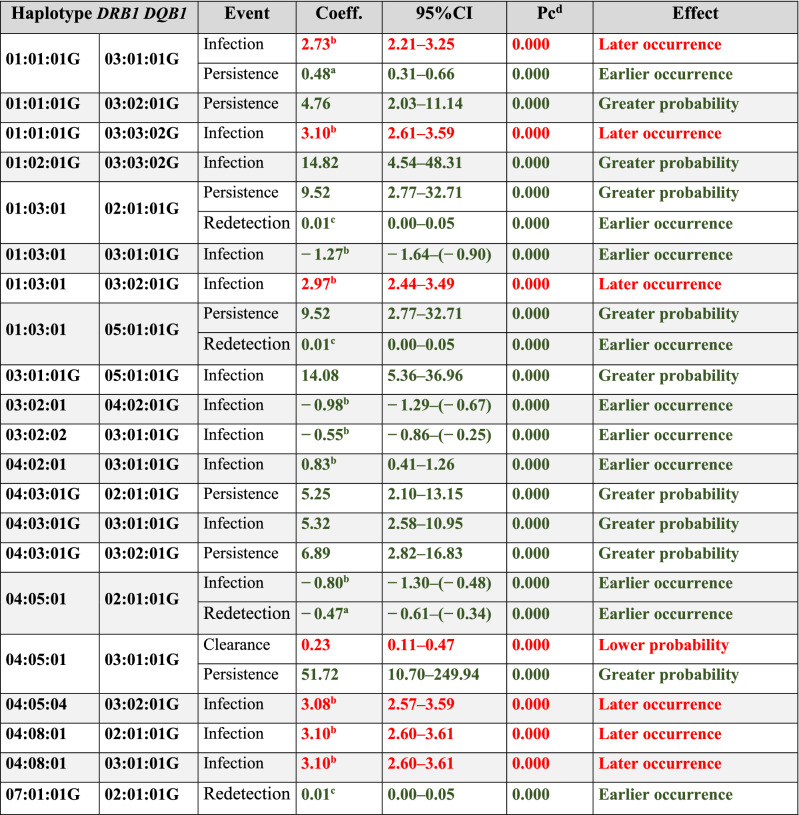

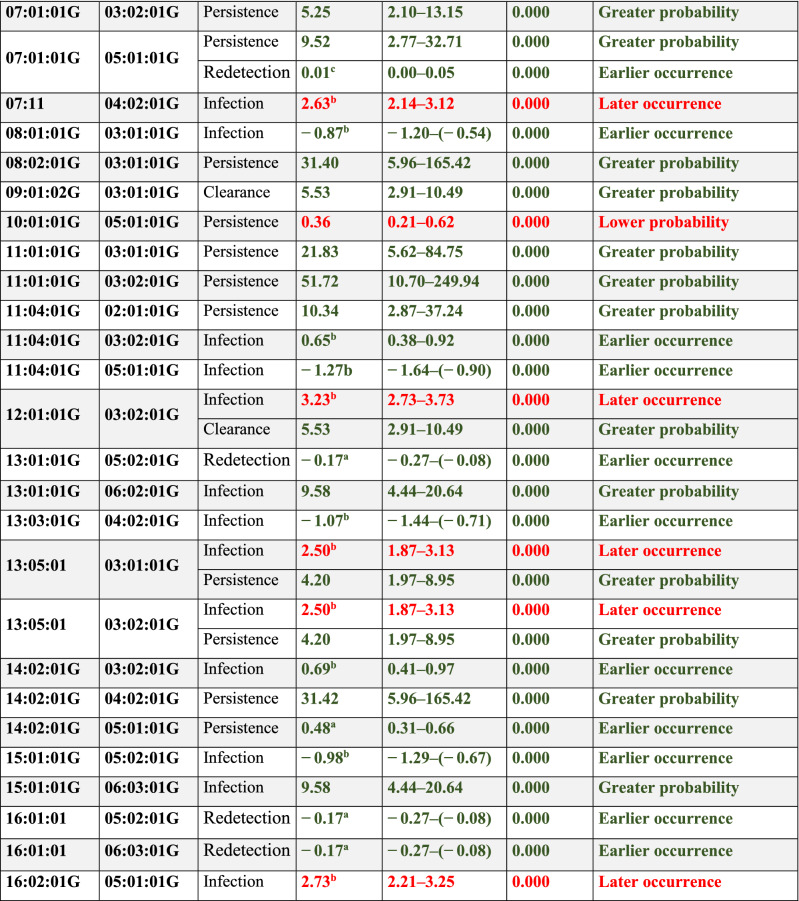
HLA *DRB1-DQB1* haplotypes associated with *Ct* infection, clearance, persistence and redetection.

Analysis was adjusted for age, age at onset of sexual life, lifetime amount of sexual partners, planning method, abortions, history of other STI and HPV infections.

Alleles affecting an event are shown as green associations [i.e. greater probability (GP) or earlier occurrence (EO)] and those hindering them [lower probability (LP) or later occurrence (LO)] as red associations.

Values in bold indicate statistical significance based on 95%CI, *p* < 0.05.

*Pc* corrected p value, *95%CI* 95% confidence interval, *Coeff* regression coefficient, *DRB1* DR beta 1, *DQB1* DQ beta 1.

^a^The Cox proportional hazards model did not fulfil the assumption of proportionality, the logistic parametric model was constructed.

^b^The Cox proportional hazards model did not fulfil the assumption of proportionality, the lognormal parametric model was constructed.

^c^The Cox proportional hazards model did not fulfil the assumption of proportionality, the Gompertz parametric model was constructed.

^d^The Bonferroni method was used for correcting all models’ *p* values.

Eighteen associations were found regarding persistence; sixteen of them related to the event (GP/EO), DRB1*04:05:01-DQB1*03:01:01G and DRB1*11:01:01G-DQB1*03:02:01G having the greatest effect. By contrast, DRB1*10:01:01G-DQB1*05:01:01G and DRB1*14:02:01G-DQB1*05:01:01G reduced persistence (LP/LO). Eight haplotypes were related to redetection events (Table 3 and Supplementary Table S8).

Nine haplotypes were related to more than one event when analysed; 6 were related to some infection events, such as EO for DRB1*04:05:01-DQB1*02:01:01G on infection and redetection, LO for DRB1*12:01:01G-DQB1*03:02:01G on infection and GP on clearance;

whilst others had an opposite effect on events, LO of DRB1*01:01:01G-DQB1*03:01:01G infection and EO on persistence (Table 3).

### In silico predicted peptides associated with *Ct* events

Peptides derived from proteins predicted as being secreted (Supplementary Table S9) were predicted in silico since it has been shown that they could be associated with protective immunity or susceptibility to *Ct* infection^7,10^. Fifteen out of 24 proteins had peptides having T-cell epitopes binding strongly to HLA-II-*DRB1* but not to -*DQB1* molecules (Supplementary Tables S10 to S24). Some peptides might have been related to increased susceptibility against *Ct* as they were associated with GP and EO effects related to persistence or redetection events. Twenty-seven peptides were related to protection (of which 7 were OMP-derived and 17 from PMP) since they were associated with effects related to *Ct* elimination (LO of infection, GP of clearance and LP of persistence) (Supplementary Tables S10 to S24). Interestingly, 11 of them had potential B-cell epitope regions, thus highlighting their possible role as cell and/or humoral immune response mediators (Table 4).

**Table 4:**
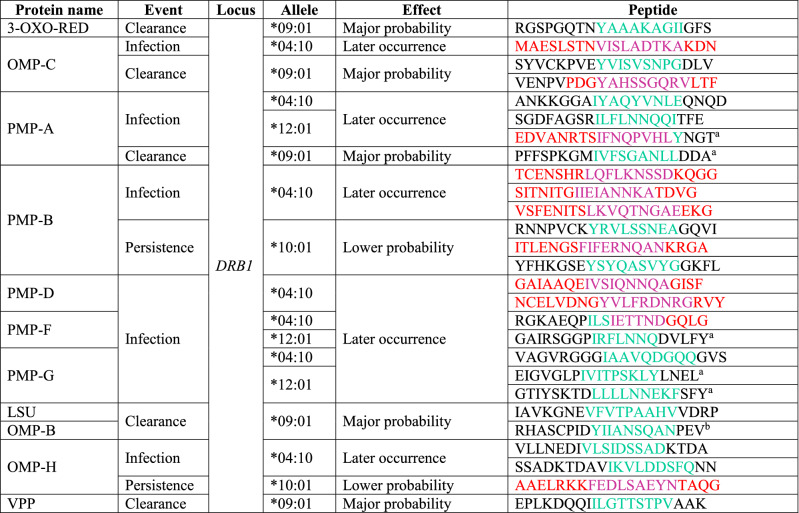
Protein regions having the potential to be T- or B-cell epitopes.

T-(green) or B-cell (red) epitope prediction is shown; regions having dual prediction are shown in purple.

^a^Peptides which could be related to GP of clearance when they are presented by the same allele.

^b^Peptides related to LP of persistence when presented by DRB1*10:01 allele.

## Discussion

*Ct* is the commonest sexually-transmitted bacterial pathogen worldwide; it can provoke serious consequences regarding reproductive sexual health once it becomes a chronic infection. Despite significant advances having been made regarding its control, clear and effective tools for reducing its impact on public health are still not available. Therefore, comprehensively evaluating a *Ct* infection dynamics-related immune response represents an alternative approach for developing effective control tools^15,16^.

This study was focused on comprehensively investigating (the first time) the effect of HLA-*DRB1*-*DQB1* alleles on *Ct* infection outcome, given that it has been shown that HLA molecules could be related to *Ct*-induced diseases, such as trachoma^17^, PID^6,18^ and infertility^19,20^, or be associated with infection prevalence and bacterial reinfection^8,21,22^. It was found that HLA-*DRB1* alleles were associated with cervical-related *Ct* infection outcome (Tables 2 and 3). Some alleles were less common for this locus and occurred at lower frequency in the target population. MHC-pathogen coevolution models indicate that less commonly occurring alleles provide greater protection against pathogens than more commonly occurring ones to which pathogens may have become adapted^23,24^.

DQB1*05:03:01G was only associated with *Ct* clearance and persistence events (Table 2); however, previous studies have reported HLA-*DQB1* (DQB1*06 and DQB1*04:02) alleles’ association with *Ct* infection and reinfection and increased bacterial persistence marker cHSP60^8,21,22^. Such discrepancy could be explained by the genetic background of the particular population being studied (African compared to South-American in this study) thereby contributing to modulating an immune response to bacterial infection^6,25^. However, these alleles only had similar associations to those reported in previous studies when configured as haplotypes, i.e. when they have been combined with a DRB1 allele (Table 3).

LP of clearance and EO of persistence were found for homozygous HLA-*DRB1* (Supplementary Tables S3 and S4). It has been reported that homozygosity is related to susceptibility to infection whilst heterozygosity is associated with a higher probability of eliminating it, possibly due to a greater immune response, given the broader amount of HLA-II restricted epitopes that can be presented to T-cells^24,26,27^. It is worth noting that findings regarding alleles causing effects on events promoting or reducing infection (Table 2) are useful when designing *Ct* infection control strategies; for example, considering peptides presented by DRB1*12:01 could represent a good strategy since it is associated with events related to infection resolution whilst peptides presented by DRB1*08:02 should be avoided as it is related to events associated with infection, such as persistence and redetection.

It has been suggested that Ag presentation during adaptative response could be an important mechanism for controlling *Ct* infection^8,28^; identifying T-cell antigens able to stimulate protection-inducing immunity is thus the key for developing anti-*Ct* vaccines^29^. Analysing *Ct* molecules’ T-cell epitopes (Supplementary Tables S10 to S24) whose role in protection-inducing immunity was experimentally evaluated^10^ revealed that 3-oxoacyl-[acyl-carrier protein] reductase had a peptide related to a GP of *Ct* clearance (Table 4). This peptide had been found in an immunoproteomics study demonstrating that inoculating dendritic cells previously pulsed with a peptide mixture (including the peptide discussed here) triggered a response partially protecting mice from intranasal and genital tract *Chlamydia* infection^30^. It has been demonstrated that CPAF-derived peptides could be related to a protection-inducing effect in a HLA-*DR4* (HLA-DRB1*04:01) transgenic mouse model^31^, however, no allele/peptide association was found in this study (Supplementary Table S12), possibly due to the allele’s low frequency in the studied population (less than 0.2%), suggesting an allele-specific effect.

The OMPs and PMPs had various regions containing T-cell epitopes, the most important ones being related to events associated with *Ct* infection elimination (Table 4). Interestingly, there was discrepancy amongst several events which could have been explained by binding core mutations for some PMP-derived peptides (PMP-B and PMP-F); these enabled discriminating between invasive (L2 and LGV) and non-invasive variants (A, B, C, D, E, F, H, J, Ja and La) (Supplementary Table S16 and S18).

It has been reported recently that the *Ct* OMP (CTH522) protein being evaluated in phase 1 trials was able to trigger a more consistent cell-mediated immune response profile after its immunisation using CAF01 liposomes compared to the placebo group, thus highlighting its potential usefulness as a vaccine candidate^32^. Such result, added to this study’s findings, supports the idea that T-cell epitopes derived from the antigens analysed here (mainly surface-derived molecules, particularly totally conserved ones) could be regions of interest for the future design of novel interventions aimed at controlling *Ct* infection.

In silico analysis suggested that some protection-related predicted peptides would specifically stimulate T-cells whilst others would stimulate both T- and B-cells (Table 4). Vaccination with various OMP serovars (D, E and F) has elicited an antibody (Ab) response neutralising bacteria in vitro^33^. Furthermore, PMPs can trigger an immune response against genital^34^ and ocular^35^ *Chlamydia* infection and a serological response in humans^36^. Mice vaccinated with DC/PMP-derived peptides (G, E and F) or with just immunogens in formulation have developed immunity against genital tract and pulmonary *Chlamydia* infection, significantly reducing bacteria in UFI assays^7,37^. Interestingly, phase 1 clinical trial vaccination using the *Ct*-OMP version showed accelerated seroconversion, increased IgG titres and enhanced mucosal profile, thus making CTH522 a promising candidate for further clinical development^32^.

As CD4 T-cells are essential for resolving primary genital infection^38^ and CD8 T-cells are important for eliminating *Ct*-infected cells by effector mechanisms^39^, then peptides stimulating both types of effector cells (B- and T), as predicted here, could be considered most suitable for controlling *Ct* infection and therefore as promising candidates for future studies (Table 4).

Antigenic peptides recognised by both CD4 and CD8 T-cells could be promising diagnostic and therapeutic tool candidates since one of the main limitations for developing an effective vaccine lies in identifying *Ct* epitopes capable of being recognised by both cell types^40^; an Ab-mediated immune response would reduce bacterial load, thereby facilitating further elimination of infection via a cell-mediated immune response^33,40^.

Considering this and given the in silico analysis performed here, it can thus be suggested that a universal anti-*Ct* vaccine should contain peptides having the following characteristics: they should induce immunity and be protein-derived, have a high degree of conservation, be associated with protection-related events (such as GP of clearance and LP of infection, redetection and persistence) and be able to stimulate T- and B-cell responses.

HLA-*DRB1*-*DQB1* alleles/haplotypes having an effect on *Ct* resolution have thus been reported here, along with in silico predicted epitopes derived from protection-related proteins targeting *Ct* infection. However, functional read-outs for demonstrating the effect of presentation regarding the predicted antigens (i.e. wet-laboratory assays) were not performed here, thus failing to obtain a complete panorama of anti-*Ct* immune responses constitutes a limitation of this study.

Addressing new prophylactic and therapeutic targets must become a high priority as the tools used to date for *Ct* control have not had a significant impact on reducing bacterial infection load. Future analysis should be aimed at validating predicted epitopes’ immunogenic and immunological in vitro and in vivo properties and their safe and efficacy regarding humans. Such data will provide relevant knowledge for understanding the usefulness of peptides as a vaccine component and the influence of host factors on the clinical course of *Ct* infection.

## Materials and methods

### Study design and participants

A cohort was studied between 2007 and 2010; that previous study was aimed at determining the natural history of HPV infection in women from the Colombian cities of Bogotá, Girardot and Chaparral; all the women were attending hospital clinics as outpatients. The study’s objective was explained to them and they voluntarily accepted participating in the study by signing an informed consent form, as described previously^13^. Retrospective analysis inclusion criteria consisted of having available cervical samples for typing HLA-*DRB1* and *DQB1* and *Ct* detection, women having attended at least four follow-up sessions (one base line and three visits) and 6-monthly periods between visits (± 3 months).

The women filled in a survey form during each visit for compiling data regarding sociodemographic information and risk factors. Such information included data regarding whether they had received/used any type of treatment between visits; none of the women reported using antibiotics during follow-up. The women did not receive antibiotic treatment for *Ct* infections detected during the study, given the retrospective nature of *Ct* detection. The Universidad del Rosario’s School of Medicine and Health Sciences Research Ethics Committee approved the study (CEI-ABN026-000135). All procedures were performed in accordance with Helsinki Declaration guidelines.

### *Ct* detection and HLA-*DRB1-DQB1 typing*

Previously obtained genomic DNA (gDNA)^13,41^ was used as template for detecting *Ct* by conventional PCR, amplifying a cryptic plasmid ORF2 segment from KL5/KL6 and KL1/KL2 primers^41^. An Illumina MiSeq (San Diego, CA, USA; Histogenetics, Ossining, NY, USA) sequencer was used for typing HLA alleles from *DRB1*-*DQB1* loci exons 2 and 3; the IPD-IMGT/HLA database (https://www.ebi.ac.uk/ipd/imgt/hla) published in January 2018 (3.31.0) was used for assigning alleles^13^.

### Statistical analysis

Qualitative variables were expressed as percentages. The Chi^2^ or Fisher’s exact tests were used for evaluating association/concordance amongst categorical variables. Continuous variables were expressed as means [with standard deviations (SD) for measure of dispersion] or medians [interquartile ranges (IQR)].

*Ct* infection was defined for this study’s purposes as PCR detection of bacterial DNA at any point during follow-up (2 years). Clearance was understood as the elimination of infection via a previous positive *Ct* result. A percentage of infections persisted before becoming eliminated; this event was evaluated in the study and was defined as *Ct* being detected during two or more consecutive follow-ups. Redetection was defined as bacterial detection after not having detected bacterial DNA during a previous follow-up.

Incidence rates for events were reported along with 95% confidence intervals (95%CI). The Kaplan–Meier estimator was used for estimating the probability of subjects continuing event-free. Cox proportional hazards models were constructed for evaluating outcome probability; such models’ coefficients were expressed as hazard rate (HR) and used for identifying alleles/haplotypes related to the evens being evaluated. Schoenfeld residuals were plotted to test the proportional hazard assumption; the covariables considered for the plot were those having *p* < 0.200 in univariate analysis (Supplementary Table S2). Variance inflation factor (VIF) and tolerance values were used for evaluating multicollinearity between covariables^13^.

Different parametric survival models were constructed when proportional hazards assumptions were not met. Akaike (AIC) and Bayesian (BIC) information criteria were used for selecting the models having the best fit. The Bonferroni method was used for correcting *p*-values for each model^13^. All two-tailed hypothesis tests (except those involved in constructing the models) were run with 0.05 significance. STATA14 software was used for analysis.

### T- and B-cell epitope prediction

*Ct* variant-derived protein amino acid (aa) sequences (Supplementary Table S25) were downloaded from the PATRIC 3.5.11 database (https://www.patricbrc.org) and analysed first using classical pathway secretion predictor (SignalP 5.0)^42^ and those not assigned by this predictor were analysed by the non-classical one, SecretomeP 2.0^43^.

The Technical University of Denmark’s Systems Biology Department’s Center for Biological Sequence Analysis’ NetMHCIIpan 3.2 server was used for assessing peptides having high predicted HLA-*DRB1*-*DQB1* allele binding activity, i.e. proteins predicted as secreted^44^. Peptides having < 2.0% rank were considered to have strong binding. The BepiPred 2.0 tool (0.6 epitope threshold) was used for calculating B-cell epitopes derived from proteins whose peptides were associated with protection^45^.

## Supplementary Information

Supplementary Information.

## Data Availability

The datasets produced and/or analysed during this study are available from the corresponding author on reasonable request.
