# Supplementary material for: Identifying HLA DRB1-DQB1 alleles associated with Chlamydia trachomatis infection and in silico prediction of potentially-related peptides
Source: Sci Rep. 2021 Jun 18;11:12837. doi: 10.1038/s41598-021-92294-w (PMC8213839; doi:10.1038/s41598-021-92294-w)
Supplement: Supplementary file 1 — Supplementary Information. [file 41598_2021_92294_MOESM1_ESM.docx]

**Identifying HLA *DRB1-DQB1* alleles associated with *Chlamydia trachomatis* infection and *in silico* prediction of potentially-related peptides**

Leidy Pedraza^1,2†^, Milena Camargo^1,3†^, Darwin A. Moreno-Pérez^1,3†^, Ricardo Sánchez^1,4^, Luisa Del Río-Ospina^1^, Indira M. Báez-Murcia^1^, Manuel E. Patarroyo ^1,4,5^, Manuel A. Patarroyo ^1,4,5*^

^1^ Molecular Biology and Immunology Department, Fundación Instituto de Inmunología de Colombia (FIDIC), Bogotá D.C. 111321, Colombia

^2^ MSc Programme in Microbiology, Universidad Nacional de Colombia, Bogotá D.C. 111321, Colombia

^3^Animal Science Faculty, Universidad de Ciencias Aplicadas y Ambientales, Bogotá D.C. 111166, Colombia

^4^Faculty of Medicine, Universidad Nacional de Colombia, Bogotá D.C. 111321, Colombia.

^5^ Health Sciences Division, Main Campus, Universidad Santo Tomás, Bogotá D.C. 110231, Colombia

^†^These authors contributed equally to this work

***Corresponding author:**

e-mail: [mapatarr.fidic@gmail.com](mailto:mapatarr.fidic@gmail.com) (MAP)

Postal address: Cra. 50 # 26-20, Bogotá D.C. 111321, Colombia

Telephone number: +57-1-3244672 ext. 141

**Supplementary Figure S1.** Workflow chart and infection, clearance, persistence and redetection rates. MT, median time; 95%CI, 95% confidence interval; FU, follow-up; Ct, *Chlamydia trachomatis*

**Supplementary Table S1.** Prevalence of HLA-DRB1 and DQB1 alleles at the local level and global classification of the common and well-documented (CWD) allele catalog.

|  |  | **This study** | | **Local** | **Global^a^** | **Ref** |
| --- | --- | --- | --- | --- | --- | --- |
|  | **Allele** | n | % |  |  |  |
| ***DRB1*** | **01:01:01G** | 18 | 3.4 | 4% allele *01:01 | Categorised as common in population groups | [^1^](#_ENREF_1)^,^[^2^](#_ENREF_2) |
|  | **01:02:01G** | 14 | 2.7 | 4% allele *01:02 | Categorised as common in population groups |  |
|  | **01:03:01** | 6 | 1.1 | 1% allele *01:03 | *01:03 categorised as common in population groups |  |
|  | **03:01:01G** | 30 | 5.7 | 5% allele *03:01 | Categorised as common in population groups |  |
|  | **03:02:01** | 2 | 0.4 | 2% allele *03:02 | Categorised as common in population groups |  |
|  | **03:02:02** | 4 | 0.8 |  |  |  |
|  | **04:01:01G** | 1 | 0.2 | 1% allele *04:01 | Categorised as common in population groups |  |
|  | **04:02:01** | 9 | 1.7 | 1% allele *04:02 | *04:02 Categorised as common in population groups |  |
|  | **04:03:01G** | 9 | 1.7 | 1% allele *04:03 | Categorised as common in population groups |  |
|  | **04:04:01** | 19 | 3.6 | 4% allele *04:04 | *04:04 categorised as common in population groups |  |
|  | **04:05:01** | 9 | 1.7 | 3% allele *04:05 | Categorised as common in population groups |  |
|  | **04:05:04** | 9 | 1.7 |  |  |  |
|  | **04:06:01G** | 1 | 0.2 | 0.1% allele *04:06 | Categorised as common in population groups |  |
|  | **04:07:01G** | 69 | 13.2 | 12% allele *04:07 | Categorised as common in population groups |  |
|  | **04:08:01** | 2 | 0.4 | 1% allele *04:02 | *04:08 Categorised as common in population groups |  |
|  | **04:10:01G** | 2 | 0.4 | 0.3% allele *04:10 | Categorised as common in population groups |  |
|  | **04:11:01** | 10 | 1.9 | 2% allele *04:11 | *04:11 Categorised as common in population groups |  |
|  | **07:01:01G** | 42 | 8.0 | 9% allele *07:01 | Categorised as common in population groups |  |
|  | **07:11** | 2 | 0.4 | 0.1% | Categorised as common in population groups |  |
|  | **08:01:01G** | 5 | 1.0 | 1% allele *08:01 | Categorised as common in population groups |  |
|  | **08:02:01G** | 27 | 5.2 | 6% allele *08:02 | Categorised as common in population groups |  |
|  | **08:03:02** | 1 | 0.2 | 0.2 % allele *08:03 | Categorised as common in population groups |  |
|  | **08:04:01** | 6 | 1.1 | 1% allele *08:04 | *08:04 categorised as common in population groups |  |
|  | **08:06** | 1 | 0.2 | 0.2% | Categorised as common in population groups |  |
|  | **08:07** | 4 | 0.8 | 0.1% | Categorised as well-documented in AF, intermediate in EU and common in AS, LA and NA |  |
|  | **09:01:02G** | 5 | 1.0 | 1% allele *09:01 | Categorised as common in population groups |  |
|  | **10:01:01G** | 9 | 1.7 | 2% allele *10:01 | Categorised as common in population groups |  |
|  | **11:01:01G** | 11 | 2.1 | 3% allele *11:01 | Categorised as common in population groups |  |
|  | **11:01:02** | 2 | 0.4 |  |  |  |
|  | **11:02:01** | 6 | 1.1 | 1% allele *11:02 | *11:02 Categorised as common in population groups |  |
|  | **11:04:01G** | 1 | 0.2 | 2% allele *11:04 | *11:04 Categorised as common in population groups |  |
|  | **11:14:01** | 13 | 2.5 | 0.1% allele *11:14 | Categorised as intermediate in EU and common in AF, AS, LA and NA |  |
|  | **12:01:01G** | 5 | 1.0 | 1% allele *12:01 | Categorised as common in population groups |  |
|  | **13:01:01G** | 28 | 5.3 | 5% allele *13:01 | Categorised as common in population groups |  |
|  | **13:02:01G** | 22 | 4.2 | 4% allele *13:02 | Categorised as common in population groups |  |
|  | **13:03:01G** | 5 | 1.0 | 1% allele *13:03 | Categorised as common in population groups |  |
|  | **13:04** | 2 | 0.4 | 0.4% | Categorised as intermediate in EU and common in AF, AS, LA and NA |  |
|  | **13:05:01** | 4 | 0.8 | 0.3% allele *13:05 | Categorised as common in population groups |  |
|  | **14:01:01G** | 8 | 1.5 | 2% allele *14:01 | Categorised as common in population groups |  |
|  | **14:02:01G** | 20 | 3.8 | 4% allele *14:02 | Categorised as common in population groups |  |
|  | **14:04:01** | 1 | 0.2 | 0.1% allele *14:04 | Categorised as common in population groups |  |
|  | **14:06:01** | 1 | 0.2 | 0.1% allele *14:06 | Categorised as common in population groups |  |
|  | **15:01:01G** | 36 | 6.9 | 6% allele *15:01 | Categorised as common in population groups |  |
|  | **15:02:01G** | 5 | 1.0 | 1% allele *15:02 | Categorised as common in population groups |  |
|  | **15:03:01G** | 3 | 0.6 | 1% allele *15:03 | Categorised as common in population groups |  |
|  | **16:01:01** | 6 | 1.1 | 0.4% allele *16:01 | *16:01 categorised as common in population groups (AF, AS, EU, LA and NA) |  |
|  | **16:02:01G** | 29 | 5.5 | 4% allele *16:02 | Categorised as common in population groups |  |
| ***DQB1*** | **01:01:01G** | 72 | 13.7 | Not documented | |  |
|  | **03:01:01G** | 107 | 20.4 | 19% allele *03:01 | Categorised as common in population groups |  |
|  | **03:02:01G** | 123 | 23.5 | 22% allele *03:02 | Categorised as common in population groups |  |
|  | **03:02:03** | 1 | 0.2 |  |  |  |
|  | **03:03:02G** | 7 | 1.3 | 2% allele *03:03 | Categorised as common in population groups |  |
|  | **04:02:01G** | 55 | 10.5 | 11% allele *04:02 | Categorised as common in population groups |  |
|  | **05:01:01G** | 61 | 11.6 | 12% allele *05:01 | Categorised as common in population groups |  |
|  | **05:02:01G** | 7 | 1.3 | 1% allele *05:02 | Categorised as common in population groups |  |
|  | **05:03:01G** | 8 | 1.5 | 2% allele *05:03 | Categorised as common in population groups |  |
|  | **06:01:01G** | 3 | 0.6 | 1% allele *06:01 | Categorised as common in population groups |  |
|  | **06:02:01G** | 33 | 6.3 | 7% allele *06:02 | Categorised as common in population groups |  |
|  | **06:03:01G** | 25 | 4.8 | 5% allele *06:03 | Categorised as common in population groups |  |
|  | **06:04:01G** | 8 | 1.5 | 2% allele *06:04 | Categorised as common in population groups |  |
|  | **06:09:01G** | 14 | 2.7 | 1% allele *06:09 | Categorised as common in population groups |  |

^a^The populations included were African, Asiatic, European, Latin-Ameican and North American

Abbreviations: Ref, Reference; DRB, DR beta 1; DQB1, DQ beta 1; AF, African; AS, Asiatic, EU, European LA, Latin-American; NA, North American

**Supplementary Table S2.** Covariable distribution regarding *Ct* outcome

|  | *C. trachomatis* outcome | | | | | | | |  |
| --- | --- | --- | --- | --- | --- | --- | --- | --- | --- |
|  | **Infection**  **(n=184)** | | **Clearance**  **(n=78)** | | **Persistence**  **(n=70)** | | **Redetection** **(n=53)** | | ***p*-value** |
| Characteristic | **n** | **%** | **n** | **%** | **n** | **%** | **n** | **%** |  |
| Age in years |  |  |  |  |  |  |  |  |  |
| *< 30* | 27 | 14.7 | 12 | 15.4 | 8 | 11.4 | 8 | 15.1 | 0.001 |
| *30-40* | 38 | 20.7 | 27 | 34.6 | 17 | 24.3 | 14 | 26.4 |  |
| *> 40* | 119 | 64.7 | 39 | 50.0 | 45 | 64.3 | 31 | 58.5 |  |
| Amount of sexual partners |  |  |  |  |  |  |  |  |  |
| *One* | 80 | 44.9 | 35 | 44.9 | 32 | 47.1 | 18 | 34.0 | 0.001 |
| *Two* | 64 | 36.0 | 20 | 25.6 | 20 | 29.4 | 17 | 32.1 |  |
| *≥ 3* | 34 | 19.1 | 23 | 29.5 | 16 | 23.5 | 18 | 34.0 |  |
| Contraceptive method |  |  |  |  |  |  |  |  |  |
| *None* | 66 | 38.4 | 27 | 38.0 | 28 | 41.8 | 20 | 41.7 | 0.165 |
| *Hormonal* | 20 | 11.6 | 6 | 8.5 | 7 | 10.4 | 6 | 12.5 |  |
| *Other ^a^* | 86 | 50.0 | 38 | 53.5 | 32 | 47.8 | 22 | 45.8 |  |
| Age at onset of sexual life |  |  |  |  |  |  |  |  |  |
| *≤ 18 years-old* | 112 | 60.9 | 42 | 53.8 | 36 | 51.4 | 31 | 58.5 | 0.001 |
| *> 18 years-old* | 72 | 39.1 | 36 | 46.2 | 34 | 48.6 | 22 | 41.5 |  |
| Abortions |  |  |  |  |  |  |  |  |  |
| *No* | 72 | 52.9 | 39 | 52.7 | 23 | 46.0 | 19 | 48.7 | 0.001 |
| *Yes* | 64 | 47.1 | 35 | 47.3 | 27 | 54.0 | 20 | 51.3 |  |
| History of other STI |  |  |  |  |  |  |  |  |  |
| *No* | 141 | 76.6 | 65 | 83.3 | 61 | 87.1 | 38 | 71.7 | 0.001 |
| *Yes* | 43 | 23.4 | 13 | 16.7 | 9 | 12.9 | 15 | 28.3 |  |
| HPV test result |  |  |  |  |  |  |  |  |  |
| *Negative* | 28 | 15.2 | 10 | 12.8 | 4 | 5.7 | 4 | 7.5 | 0.001 |
| *Positive* | 156 | 84.8 | 68 | 87.2 | 66 | 94.3 | 49 | 92.5 |  |

^a^ Contraceptive method category ‘other’ included barrier methods and surgery

STI, sexually-transmitted infection

**Supplementary Table S3.** HLA-*DRB1* alleles associated with *Ct* infection and clearance

| **Allele** | **Infection** | | | |  | **Clearance** | | | |
| --- | --- | --- | --- | --- | --- | --- | --- | --- | --- |
|  | **HR** | **95%CI** | ***p*** | **Pc^b^** |  | **HR** | **95%CI** | ***p*** | **Pc^b^** |
| ***DRB1*** |  |  |  |  |  |  |  |  |  |
| **01:01:01G** | 0.24^a^ | -0.17-0.66 | 0.247 | . |  | 0.94 | 0.64-1.39 | 0.791 | . |
| **01:02:01G** | **8.36** | **1.71-4.08** | **0.000** | **0.000** |  | 0.27 | 0.09-0.75 | **0.013** | 0.442 |
| **01:03:01** | -0.03^a^ | -0.81-0.75 | 0.937 | . |  | 1.71 | 0.90-3.22 | 0.096 | . |
| **03:01:01G** | 0.18^a^ | -0.23-0.61 | 0.378 | . |  | 0.55 | 0.33-0.92 | **0.024** | 0.816 |
| **03:02:01** | **-0.98**^a^ | **-1.29-(-0.67)** | **0.000** | **0.000** |  | 0.74 | 0.43-1.26 | 0.272 | . |
| **03:02:02** | **-0.55^a^** | **-0.86-(-0.25)** | **0.000** | **0.000** |  | 0.48 | 0.30-0.76 | **0.002** | 0.068 |
| **04:01:01G** | 0.00^a^ | . | . | . |  | NA | . | . | . |
| **04:02:01** | 0.07^a^ | -0.50-0.66 | 0.798 | . |  | NA | . | . | . |
| **04:03:01G** | 2.86 | 0.80-10.13 | 0.103 | . |  | NA | . | . | . |
| **04:04:01** | NA | . | . | . |  | NA | . | . | . |
| **04:05:01** | 2.37 | 0.81-6.92 | 0.112 | . |  | 0.64 | 0.36-1.13 | 0.127 | . |
| **04:05:04** | 1.18 | 0.34-4.12 | 0.787 | . |  | 1.02 | 0.26-4.02 | 0.969 | . |
| **04:06:01G** | 1.02 | 0.18-5.74 | 0.978 | . |  | NA | . | . | . |
| **04:07:01G** | 0.94 | 0.57-1.56 | 0.836 | . |  | 1.60 | 1.07-2.37 | **0.020** | 0.680 |
| **04:08:01** | 0.85 | 0.39-1.98 | 0.716 | . |  | 0.41 | 0.19-0.88 | **0.022** | 0.748 |
| **04:10:01G** | **2.91^a^** | **2.49-3.36** | **0.000** | **0.000** |  | NA | . | . | . |
| **04:11:01** | -0.35^a^ | -0.62-(-0.08) | **0.011** | 0.495 |  | 0.92 | 0.57-1.51 | 0.764 | . |
| **07:01:01G** | -0.11^a^ | -0.40-0.16 | 0.419 | . |  | 0.78 | 0.38-1.60 | 0.512 | . |
| **07:11** | 0.29^a^ | -0.17-0.76 | 0.224 | . |  | NA | . | . | . |
| **08:01:01G** | **-0.87^a^** | **-1.20-(-0.54)** | **0.000** | **0.000** |  | NA | . | . | . |
| **08:02:01G** | -0.41^a^ | -0.95-0.12 | 0.129 | . |  | 0.68 | 0.27-1.69 | 0.416 | . |
| **08:03:02** | 0.00^a^ | . | . | . |  | 1.11 | 0.54-2.25 | 0.768 | . |
| **08:04:01** | 0.63^a^ | 0.09-1.16 | **0.020** | 0.900 |  | 0.92 | 0.53-1.60 | 0.785 | . |
| **08:06** | 1.59 | 0.46-5.49 | 0.460 | . |  | NA | . | . | . |
| **08:07** | 1.00 | . | . | . |  | 6.45 | 1.27-32.75 | **0.024** | 0.816 |
| **09:01:02G** | -0.34^a^ | -0.80-0.11 | 0.139 | . |  | **5.53** | **2.91-10.49** | **0.000** | **0.000** |
| **10:01:01G** | 0.73 | 0.09-5.66 | 0.765 | . |  | 0.85 | 0.50-1.45 | 0.565 | . |
| **11:01:01G** | 1.02 | 0.48-2.14 | 0.955 | . |  | 1.02 | 0.54-1.91 | 0.948 | . |
| **11:01:02** | **3.00^a^** | **2.48-3.51** | **0.000** | **0.000** |  | 1.52 | 0.40-5.66 | 0.531 | . |
| **11:02:01** | 0.70 | 0.18-2.62 | 0.601 | . |  | 1.34 | 0.68-2.64 | 0.385 | . |
| **11:04:01G** | -0.11^a^ | -0.45-0.22 | 0.511 | . |  | 1.32 | 0.69-2.52 | 0.388 | . |
| **11:14:01** | 0.25^a^ | -0.10-0.61 | 0.170 | . |  | NA | . | . | . |
| **12:01:01G** | **3.23^a^** | **2.73-3.73** | **0.000** | **0.000** |  | **5.53** | **2.91-10.49** | **0.000** | **0.000** |
| **13:01:01G** | 0.44^a^ | 0.03-0.85 | **0.032** | 1.440 |  | 0.82 | 0.50-1.32 | 0.420 | . |
| **13:02:01G** | 1.40 | 0.67-2.90 | 0.361 | . |  | 1.82 | 0.69-4.73 | 0.220 | . |
| **13:03:01G** | -0.10^a^ | -1.46-1.24 | 0.875 | . |  | 1.24 | 0.67-2.31 | 0.483 | . |
| **13:04** | 4.87 | 1.30-18.27 | **0.019** | 0.855 |  | NA | . | . | . |
| **13:05:01** | NA | . | . | . |  | 0.83 | 0.55-1.27 | 0.414 | . |
| **14:01:01G** | -0.07^a^ | -0.57-0.42 | 0.768 | . |  | 4.97 | 1.79-13.74 | **0.002** | 0.068 |
| **14:02:01G** | **2.40^a^** | **1.87-3.10** | **0.000** | **0.000** |  | 1.51 | 0.87-2.62 | 0.140 | . |
| **14:04:01** | 0.00^a^ | . | . | . |  | NA | . | . | . |
| **14:06:01** | 0.00^a^ | . | . | . |  | NA | . | . | . |
| **15:01:01G** | 1.33 | 0.49-3.57 | 0.569 | . |  | 1.05 | 0.61-1.79 | 0.848 | . |
| **15:02:01G** | 0.39^a^ | -0.10-0.88 | 0.120 | . |  | 3.67 | 1.34-10.04 | **0.011** | 0.374 |
| **15:03:01G** | 0.10^a^ | -0.69-0.90 | 0.792 | . |  | 1.00 | . | . | . |
| **16:01:01** | 2.77 | 0.83-9.27 | 0.097 | . |  | 1.23 | 0.55-2.76 | 0.608 | . |
| **16:02:01G** | 1.29 | 0.71-2.34 | 0.395 | . |  | 0.54 | 0.31-0.96 | **0.038** | 1.292 |
| **Homozygosity** | -0.27^a^ | -0.57-0.51 | 0.922 | . |  | **0.58** | **0.37-0.52** | **0.000** | **0.000** |

^a^ The Cox proportional hazards model did not fulfil the assumption of proportionality; a lognormal parametric model was constructed

^b^ The Bonferroni method was used for correcting all models’ *p*-values

Analysis was adjusted for age, age at onset of sexual life, lifetime amount of sexual partners, family planning method, abortions and HPV infection

Alleles affecting an event are shown as green (greater probability (GP) or earlier occurrence (EO)) or red associations (lower probability (LP) or later occurrence (LO))

Pc, corrected *p*-value; NA, not applicable; 95%CI, 95% confidence interval; Coeff, regression coefficient; *DRB, DR beta 1; DQB1, DQ beta 1*

Values in bold indicate statistical significance based on 95%CI, *p*<0.05

**Supplementary Table S4.** HLA-*DRB1* alleles associated with *Ct* persistence and redetection

| **Allele** | **Persistence** | | | |  | **Redetection** | | | |
| --- | --- | --- | --- | --- | --- | --- | --- | --- | --- |
|  | **HR** | **95%CI** | ***p*** | **Pc^d^** |  | **HR** | **95%CI** | ***p*** | **Pc^d^** |
| ***DRB1*** |  |  |  |  |  |  |  |  |  |
| **01:01:01G** | 1.40 | 0.72-2.72 | 0.314 | . |  | 14.51^b^ | 2.48-84.62 | **0.003** | 0.090 |
| **01:02:01G** | 1.27 | 0.56-2.83 | 0.558 | . |  | 0.00^a^ | . | . | . |
| **01:03:01** | 4.25 | 1.37-13.12 | **0.012** | 0.384 |  | **0.01^c^** | **0.00-0.05** | **0.000** | **0.000** |
| **03:01:01G** | 0.31 | 0.68-1.40 | 0.129 | . |  | 16.88^b^ | 1.55-183.05 | **0.020** | 0.600 |
| **03:02:01** | NA | . | . | . |  | -0.07^a^ | -0.20-0.05 | 0.237 | . |
| **03:02:02** | 0.82 | 0.46-1.46 | 0.506 | . |  | NA | . | . | . |
| **04:01:01G** | NA | . | . | . |  | NA | . | . | . |
| **04:02:01** | NA | . | . | . |  | NA | . | . | . |
| **04:03:01G** | NA | . | . | . |  | NA | . | . | . |
| **04:04:01** | NA | . | . | . |  | NA | . | . | . |
| **04:05:01** | 1.09 | 0.46-2.60 | 0.838 | . |  | -0.09^a^ | -0.52-0.34 | 0.672 | . |
| **04:05:04** | 1.43 | 0.74-2.73 | 0.277 | . |  | 5.86^b^ | 0.91-37.68 | 0.062 | . |
| **04:06:01G** | NA | . | . | . |  | NA | . | . | . |
| **04:07:01G** | 1.10 | 0.63-1.91 | 0.723 | . |  | 0.08^a^ | 0.02-0.14 | **0.007** | 0.210 |
| **04:08:01** | 0.73 | 0.36-1.48 | 0.384 | . |  | NA | . | . | . |
| **04:10:01G** | NA | . | . | . |  | NA | . | . | . |
| **04:11:01** | 0.61 | 0.33-1.14 | 0.123 | . |  | 0.10^b^ | 0.01-0.82 | **0.032** | 0.960 |
| **07:01:01G** | 1.52 | 0.35-6.62 | 0.570 | . |  | 0.03^c^ | 0.00-0.31 | **0.002** | 0.060 |
| **07:11** | 1.03 | 0.47-2.23 | 0.935 | . |  | NA | . | . | . |
| **08:01:01G** | 1.53 | 0.47-4.98 | 0.473 | . |  | -0.17^a^ | -0.36-0.02 | 0.081 | . |
| **08:02:01G** | **12.71** | **4.35-37.09** | **0.000** | **0.000** |  | **-0.32^a^** | **-0.51--(-0.13)** | **0.001** | **0.030** |
| **08:03:02** | NA | . | . | . |  | 0.00^a^ | . | . | . |
| **08:04:01** | 2.50 | 1.33-4.72 | **0.004** | 0.128 |  | NA | . | . | . |
| **08:06** | NA | . | . | . |  | 0.00^a^ | . | . | . |
| **08:07** | NA | . | . | . |  | 0.03^a^ | -0.09-0.15 | 0.626 | . |
| **09:01:02G** | 1.69 | 0.87-3.31 | 0.120 | . |  | 0.03a | -0.05-0.12 | 0.425 | . |
| **10:01:01G** | **0.33** | **0.19-0.57** | **0.000** | **0.000** |  | -0.10^a^ | -0.23-0.02 | 0.122 | . |
| **11:01:01G** | **22.20** | **5.70-86.49** | **0.000** | **0.000** |  | 2.42^b^ | 0.48-12.10 | 0.279 | . |
| **11:01:02** | NA | . | . | . |  | 0.00^a^ | . | . | . |
| **11:02:01** | 0.84 | 0.35-1.98 | 0.696 | . |  | 0.01^a^ | -0.08-0.11 | 0.792 | . |
| **11:04:01G** | 2.81 | 1.10-7.18 | **0.031** | 0.992 |  | 3.52^b^ | 0.68-18.05 | 0.131 | . |
| **11:14:01** | NA | . | . | . |  | NA | . | . | . |
| **12:01:01G** | NA | . | . | . |  | 0.03^a^ | -0.05-0.12 | 0.425 | . |
| **13:01:01G** | 0.52 | 0.27-1.01 | 0.054 | . |  | -0.02^a^ | -0.13-0.08 | 0.662 | . |
| **13:02:01G** | 0.54 | 0.22-1.36 | 0.196 | . |  | 0.10^a^ | -0.04-0.25 | 0.187 | . |
| **13:03:01G** | 2.24 | 0.65-7.68 | 0.198 | . |  | NA | . | . | . |
| **13:04** | 1.39 | 0.72-2.70 | 0.319 | . |  | NA | . | . | . |
| **13:05:01** | **3.67** | **1.74-7.75** | **0.001** | **0.032** |  | -0.02^a^ | -0.10-0.05 | 0.567 | . |
| **14:01:01G** | **3.92** | **1.86-8.25** | **0.000** | **0.000** |  | -0.02^a^ | -0.25-0.21 | 0.843 | . |
| **14:02:01G** | **27.79** | **5.30-145.59** | **0.000** | **0.000** |  | 0.00^a^ | -0.90-0.91 | 0.988 | . |
| **14:04:01** | NA | . | . | . |  | NA | . | . | . |
| **14:06:01** | 1.00 | . | . | . |  | NA | . | . | . |
| **15:01:01G** | 0.64 | 0.34-1.18 | 0.160 | . |  | 6.36^b^ | 1.77-22.76 | **0.004** | 0.120 |
| **15:02:01G** | 1.04 | 0.60-1.80 | 0.861 | . |  | NA | . | . | . |
| **15:03:01G** | NA | . | . | . |  | NA | . | . | . |
| **16:01:01** | 2.07 | 0.98-4.36 | 0.055 | . |  | **-0.17^a^** | **-0.27-(-0.08)** | **0.000** | **0.000** |
| **16:02:01G** | 1.28 | 0.48-3.38 | 0.613 | . |  | 0.01^a^ | -0.06-0.08 | 0.798 | . |
| **Homozygosity** | **0.20^a^** | **0.05-0.36** | **0.000** | **0.000** |  | NA |  |  |  |

^a^ The Cox proportional hazards model did not fulfil the assumption of proportionality; a logistic parametric model was constructed

^b^ The Cox proportional hazards model did not fulfil the assumption of proportionality; a Weibull parametric regression model for survival data was constructed

^c^ The Cox proportional hazards model did not fulfil the the assumption of proportionality; a Gompertz parametric proportional hazards model was constructed

^d^ The Bonferroni method was used for correcting the *p*-values for each model

Analysis was adjusted for age, age at onset of sexual life, lifetime amount of sexual partners, family planning method, abortions and HPV infection

Alleles affecting an event are shown in green for greater probability (GP) or earlier occurrence (EO) and red for lower probability (LP) or later occurrence (LO)

Pc, corrected *p*-value; NA, not applicable; 95%CI, 95% confidence interval; Coeff, regression coefficient; *DRB, DR beta 1; DQB1, DQ beta 1*

Values in bold indicate statistical significance based on 95%CI, *p*<0.05

**Supplementary Table S5.** HLA-*DQB1* alleles associated with *Ct* infection and clearance

| **Allele** | **Infection** | | | |  | **Clearance** | | | |
| --- | --- | --- | --- | --- | --- | --- | --- | --- | --- |
|  | **HR** | **95%CI** | ***p*** | **Pc^b^** |  | **HR** | **95%CI** | ***p*** | **Pc^b^** |
| ***DQB1*** |  |  |  |  |  |  |  |  |  |
| **02:01:01G** | 0.01^a^ | -0.22-0.25 | 0.911 | . |  | 0.57 | 0.37-0.89 | **0.015** | 0.180 |
| **03:01:01G** | 0.93 | 0.59-1.45 | 0.752 | . |  | 0.77 | 0.52-1.13 | 0.187 | . |
| **03:02:01G** | 0.02^a^ | -0.19-0.25 | 0.796 | . |  | 0.94 | 0.68-1.31 | 0.745 | . |
| **03:02:03** | NA | . | . | . |  | 1.72 | 0.47-6.19 | 0.407 | . |
| **03:03:02G** | -0.25^a^ | -0.68-0.18 | 0.252 | . |  | NC | . | . | . |
| **04:02:01G** | -0.27^a^ | -0.56-0.00 | 0.051 | . |  | 1.087 | 0.65-1.79 | 0.757 | . |
| **05:01:01G** | 0.76 | 0.42-1.35 | 0.353 | . |  | 0.97 | 0.70-1.34 | 0.859 | . |
| **05:02:01G** | 3.70 | 1.27-10.76 | **0.016** | 0.208 |  | 1.55 | 0.66-3.64 | 0.307 | . |
| **05:03:01G** | -0.07^a^ | -0.57-0.42 | 0.768 | . |  | **4.97** | **1.79-13.74** | **0.002** | **0.024** |
| **06:01:01G** | 0.30^a^ | -0.10-0.88 | 0.120 | . |  | 3.67 | 1.34-10.04 | **0.011** | 0.132 |
| **06:02:01G** | 0.88 | 0.38-2.05 | 0.777 | . |  | 0.97 | 0.56-1.67 | 0.927 | . |
| **06:03:01G** | 0.25^a^ | -0.15-0.65 | 0.218 | . |  | 0.82 | 0.50-1.32 | 0.420 | . |
| **06:04:01G** | -0.27^a^ | -0.74-0.18 | 0.241 | . |  | 1.00 | . | . | . |
| **06:09:01G** | 0.30^a^ | -0.23-0.84 | 0.272 | . |  | 1.82 | 0.69-4.73 | 0.220 | . |
| **Homozygosity** | 0.57^a^ | -0.25-0.37 | 0.721 |  |  | 1.30 | 0.80-2.11 | 0.279 |  |

^a^ The Cox proportional hazards model did not fulfil the assumption of proportionality; a lognormal parametric model was constructed

^b^ The Bonferroni method was used for correcting the *p*-values for each model

Analysis was adjusted for age, age at onset of sexual life, lifetime amount of sexual partners, family planning method, abortions and HPV infection

Alleles affecting an event are shown in green for greater probability (GP) or earlier occurrence (EO) or red for lower probability (LP) or later occurrence (LO)

Pc, corrected *p*-value; NC not calculated; NA, not applicable; 95%CI, 95% confidence interval; Coeff, regression coefficient; *DRB, DR beta 1; DQB1, DQ beta 1*

Values in bold indicate statistical significance based on 95%CI, *p*<0.05

**Supplementary Table S6**. HLA-*DQB1* alleles associated with *Ct* persistence and redetection

| **Allele** | **Persistence** | | | |  | **Redetection** | | | |
| --- | --- | --- | --- | --- | --- | --- | --- | --- | --- |
|  | **HR** | **95%CI** | ***p*** | **Pc^d^** |  | **HR** | **95%CI** | ***p*** | **Pc^d^** |
| **DQB1** |  |  |  |  |  |  |  |  |  |
| **02:01:01G** | 1.43 | 0.64-3.19 | 0.376 | . |  | -0.06^a^ | -0.33-0.20 | 0.640 | . |
| **03:01:01G** | 1.73 | 1.04-2.87 | **0.032** | 0.416 |  | 1.93^b^ | 0.93-3.98 | 0.075 | . |
| **03:02:01G** | 1.02 | 0.67-1.57 | 0.893 | . |  | 0.09a | 0.01-0.16 | **0.012** | 0.144 |
| **03:02:03** | NA | . | . | . |  | 0.04^a^ | -0.08-0.17 | 0.532 | . |
| **03:03:02G** | 1.69 | 0.87-3.31 | 0.120 | . |  | 0.08^a^ | -0.03-0.20 | 0.155 | . |
| **04:02:01G** | 1.08 | 0.57-2.05 | 0.798 | . |  | -0.03^a^ | -0.11-0.05 | 0.458 | . |
| **05:01:01G** | 0.94 | 0.53-1.67 | 0.839 | . |  | -0.05^a^ | -0.20-0.10 | 0.530 | . |
| **05:02:01G** | 2.07 | 0.98-4.36 | 0.055 | . |  | -0.12^a^ | -0.21-(-0.03) | **0.006** | 0.078 |
| **05:03:01G** | **3.92** | **1.86-8.25** | **0.000** | **0.000** |  | -0.02^a^ | -0.25-0.21 | 0.843 | . |
| **06:01:01G** | 1.04 | 0.60-1.80 | 0.861 | . |  | NA | . | . | . |
| **06:02:01G** | 0.64 | 0.34-1.18 | 0.160 | . |  | 7.46^b^ | 1.58-35.20 | **0.011** | 0.132 |
| **06:03:01G** | 0.52 | 0.27-1.01 | 0.054 | . |  | -0.02^a^ | -0.13-0.08 | 0.662 | . |
| **06:04:01G** | 0.44 | 0.15-1.26 | 0.128 | . |  | 0.00^a^ | . | . | . |
| **06:09:01G** | 1.05 | 0.45-2.43 | 0.894 | . |  | 0.10^a^ | -0.04-0.25 | 0.187 | . |
| **Homozygosity** | 0.54 | 0.26-1.12 | 0.101 | . |  | 0.49^b^ | 0.10-2.23 | 0.358 | . |

^a^ The Cox proportional hazards model did not fulfil the assumption of proportionality; a logistic parametric model was constructed

^b^ The Cox proportional hazards model did not fulfil the assumption of proportionality, a Weibull parametric model was constructed

^c^ The Cox proportional hazards model did not fulfil the assumption of proportionality, a Gompertz parametric model was constructed

^d^ The Bonferroni method was used for correcting *p*-values for all models

Analysis was adjusted for age, age at onset of sexual life, lifetime amount of sexual partners, family planning method, abortions and HPV infection.

Alleles affecting an event shown in green concern greater probability (GP) or earlier occurrence (EO) or in red for lower probability (LP) or later occurrence (LO)

Pc, corrected *p*-values; NC, not calculated; NA, not applicable; 95%CI, 95% confidence interval; Coeff, regression coefficient; *DRB, DR beta 1; DQB1, DQ beta 1*

Values in bold indicate statistical significance based on 95%CI, *p*<0.05

**Supplementary Table S7.** HLA *DRB1-DQB1* haplotypes associated with *Ct* infection and clearance

| **Haplotype** | | **Infection** | | | |  | **Clearance** | | | |
| --- | --- | --- | --- | --- | --- | --- | --- | --- | --- | --- |
|  |  | **HR** | **95%CI** | ***p*** | **Pc^b^** |  | **HR** | **95%CI** | ***p*** | **Pc^b^** |
| ***DRB1*** | ***-DQB1*** |  |  |  |  |  |  |  |  |  |
| **01:01:01G** | **-03:01:01G** | **2.73^a^** | **2.21-3.25** | **0.000** | **0.000** |  | 0.77 | 0.46-1.27 | 0.309 | . |
| **01:01:01G** | **-03:02:01G** | -0.16^a^ | -0.42-0.09 | 0.218 | . |  | 1.00 | . | . | . |
| **01:01:01G** | **-03:03:02G** | **3.10^a^** | **2.61-3.59** | **0.000** | **0.000** |  | 1.00 | . | . | . |
| **01:01:01G** | **-04:02:01G** | NA | . | . | . |  | 0.34 | 0.11-1.00 | 0.051 | . |
| **01:01:01G** | **-05:01:01G** | 0.38^a^ | -0.02-0.80 | 0.069 | . |  | 0.80 | 0.55-1.18 | 0.276 | . |
| **01:02:01G** | **-02:01:01G** | -0.18^a^ | -0.93-0.57 | 0.638 | . |  | 1.00 | . | . | . |
| **01:02:01G** | **-03:01:01G** | NA | . | . | . |  | 1.13 | 0.62-2.08 | 0.675 | . |
| **01:02:01G** | **-03:02:01G** | 0.19^a^ | -0.34-0.73 | 0.476 | . |  | 1.00 | . | . | . |
| **01:02:01G** | **-03:03:02G** | **14.82** | **4.54-48.31** | **0.000** | **0.000** |  | 1.00 | . | . | . |
| **01:02:01G** | **-04:02:01G** | 0.00^a^ | . | . | . |  | 1.00 | . | . | . |
| **01:02:01G** | **-05:01:01G** | 0.20^a^ | -0.16-0.58 | 0.282 | . |  | 1.13 | 0.62-2.08 | 0.675 | . |
| **01:03:01** | **-02:01:01G** | NA | . | . | . |  | 1.53 | 0.74-3.17 | 0.245 | . |
| **01:03:01** | **-03:01:01G** | **-1.27^a^** | **-1.64-(-0.90)** | **0.000** | **0.000** |  | 1.24 | 0.67-2.31 | 0.483 | . |
| **01:03:01** | **-03:02:01G** | **2.97^a^** | **2.44-3.49** | **0.000** | **0.000** |  | 1.00 | . | . | . |
| **01:03:01** | **-04:02:01G** | 2.76 | 1.22-6.25 | **0.014** | 1.736 |  | 1.00 | .. | . | . |
| **01:03:01** | **-05:01:01G** | 0.62 | 0.11-3.40 | 0.587 | . |  | 1.31 | 0.86-2.00 | 0.203 | . |
| **01:03:01** | **-05:02:01G** | NA | . | . | . |  | 1.00 | . | . | . |
| **03:01:01G** | **-02:01:01G** | 0.18^a^ | -0.23-0.61 | 0.378 | . |  | 0.57 | 0.35-0.91 | **0.019** | 1.976 |
| **03:01:01G** | **-05:01:01G** | **14.08** | **5.36-36.96** | **0.000** | **0.000** |  | 1.00 | . | . | . |
| **03:02:01** | **-02:01:01G** | 0.00^a^ | . | . | . |  | 1.00 | . | . | . |
| **03:02:01** | **-04:02:01G** | **-0.98^a^** | **-1.29-(-0.67)** | **0.000** | **0.000** |  | 1.00 | . | . | . |
| **03:02:02** | **-02:01:01G** | -0.60^a^ | -1.07-(-0.14) | **0.010** | 1.240 |  | 1.00 | . | . | . |
| **03:02:02** | **-03:01:01G** | **-0.55^a^** | **-0.86-(-0.25)** | **0.000** | **0.000** |  | 0.48 | 0.30-0.76 | **0.002** | 0.208 |
| **04:01:01G** | **-03:02:01G** | 0.00^a^ | . | . | . |  | 1.00 | . | . | . |
| **04:02:01** | **-02:01:01G** | 0.21^a^ | -0.15-0.58 | 0.248 | . |  | 1.00 | . | . | . |
| **04:02:01** | **-03:01:01G** | **0.83^a^** | **0.41-1.26** | **0.000** | **0.000** |  | 1.00 | . | . | . |
| **04:02:01** | **-03:02:01G** | 0.09^a^ | -0.41-0.61 | 0.711 | . |  | 1.00 | . | . | . |
| **04:02:01** | **-05:01:01G** | 0.00^a^ | . | . | . |  | 1.00 | . | . | . |
| **04:03:01G** | **-02:01:01G** | 1.46 | 0.10-20.24 | 0.776 | . |  | 1.00 | . | . | . |
| **04:03:01G** | **-03:01:01G** | **5.32** | **2.58-10.95** | **0.000** | **0.000** |  | 1.00 | . | . | . |
| **04:03:01G** | **-03:02:01G** | 5.72 | 2.01-16.28 | **0.001** | 0.124 |  | 1.00 | . | . | . |
| **04:04:01** | **-02:01:01G** | 0.50^a^ | 0.16-0.84 | **0.004** | 0.496 |  | 1.00 | . | . | . |
| **04:04:01** | **-03:01:01G** | 0.59 | 0.17-2.06 | 0.415 | . |  | 1.00 | . | . | . |
| **04:04:01** | **-03:02:01G** | 0.49^a^ | 0.13-0.86 | **0.007** | 0.868 |  | 1.46 | 0.77-2.77 | 0.237 | . |
| **04:04:01** | **-05:01:01G** | -0.04^a^ | -0.29-0.20 | 0.746 | . |  | 1.00 | . | . | . |
| **04:05:01** | **-02:01:01G** | **-0.80^a^** | **-1.30-(-0.48)** | **0.000** | **0.000** |  | 1.00 | . | . | . |
| **04:05:01** | **-03:02:01G** | 3.18 | 1.04-9.64 | **0.041** | 5.084 |  | 0.64 | 0.36-1.13 | 0.127 | . |
| **04:05:01** | **-03:01:01G** | 0.02^a^ | -0.26-0.31 | 0.845 | . |  | **0.23** | **0.11-0.47** | **0.000** | **0.000** |
| **04:05:01** | **-05:01:01G** | NA | . | . | . |  | NA | . | . | . |
| **04:05:04** | **-02:01:01G** | 0.06^a^ | -0.18-0.31 | 0.605 | . |  | 0.49 | 0.27-0.89 | **0.021** | 2.184 |
| **04:05:04** | **-03:01:01G** | 1.33 | 0.73-2.41 | 0.345 | . |  | NA | . | . | . |
| **04:05:04** | **-03:02:01G** | **3.08^a^** | **2.57-3.59** | **0.000** | **0.000** |  | 1.00 | . | . | . |
| **04:05:04** | **-04:02:01G** | 0.81 | 0.28-2.29 | 0.693 | . |  | 1.02 | 0.26-4.02 | 0.969 | . |
| **04:05:04** | **-05:01:01G** | 2.76 | 1.22-6.25 | **0.014** | 1.736 |  | NA | . | . | . |
| **04:07:01G** | **-02:01:01G** | 0.05^a^ | -0.38-0.49 | 0.800 | . |  | 0.74 | 0.43-1.26 | 0.272 | . |
| **04:07:01G** | **-03:01:01G** | 0.11^a^ | -0.30-0.53 | 0.598 | . |  | 0.66 | 0.46-0.93 | **0.019** | 1.976 |
| **04:07:01G** | **-03:02:01G** | 0.93 | 0.57-1.54 | 0.806 | . |  | 1.53 | 1.02-2.29 | **0.039** | 4.056 |
| **04:07:01G** | **-03:02:03** | NA | . | . | . |  | 1.63 | 0.51-5.19 | 0.401 | . |
| **04:07:01G** | **-04:02:01G** | -0.07^a^ | -1.24-1.08 | 0.896 | . |  | 1.52 | 0.69-3.34 | 0.293 | . |
| **04:07:01G** | **-05:01:01G** | 1.13 | 0.42-3.05 | 0.805 | . |  | 2.68 | 1.25-5.74 | **0.011** | 1.144 |
| **04:08:01** | **-02:01:01G** | **3.10^a^** | **2.60-3.61** | **0.000** | **0.000** |  | 0.32 | 0.14-0.72 | **0.006** | 0.624 |
| **04:08:01** | **-03:01:01G** | **3.10^a^** | **2.60-3.61** | **0.000** | **0.000** |  | 0.32 | 0.14-0.72 | **0.006** | 0.624 |
| **04:10:01G** | **-04:02:01G** | 1.24 | . | . | . |  | NA | . | . | . |
| **04:11:01** | **-02:01:01G** | -0.35^a^ | -0.62-(-0.08) | **0.011** | 1.364 |  | NA | . | . | . |
| **04:11:01** | **-03:02:01G** | -0.35^a^ | -0.62-(-0.08) | **0.011** | 1.364 |  | 0.92 | 0.57-1.51 | 0.764 | . |
| **04:11:01** | **-04:02:01G** | NA | . | . | . |  | 0.72 | 0.41-1.25 | 0.252 | . |
| **07:01:01G** | **-02:01:01G** | -0.02^a^ | -0.31-0.26 | 0.863 | . |  | 0.55 | 0.32-0.96 | **0.038** | 3.952 |
| **07:01:01G** | **-03:01:01G** | -0.06^a^ | -0.59-0.46 | 0.812 | . |  | NA | . | . | . |
| **07:01:01G** | **-03:02:01G** | 1.31 | 0.49-3.50 | 0.582 | . |  | 1.00 | . | . | . |
| **07:01:01G** | **-03:03:02G** | -0.11^a^ | -0.71-0.48 | 0.706 | . |  | NC | . | . | . |
| **07:01:01G** | **-04:02:01G** | 0.06^a^ | -0.18-0.31 | 0.605 | . |  | 1.19 | 0.20-6.87 | 0.839 | . |
| **07:01:01G** | **-05:01:01G** | 0.10^a^ | -0.60-0.81 | 0.770 | . |  | 1.53 | 0.74-3.17 | 0.245 | . |
| **07:11** | **-03:02:01G** | -0.35^a^ | -0.62-(-0.08) | **0.011** | 1.364 |  | NA | . | . | . |
| **07:11** | **-04:02:01G** | **2.63^a^** | **2.14-3.12** | **0.000** | **0.000** |  | NA | . | . | . |
| **08:01:01G** | **-03:01:01G** | **-0.87^a^** | **-1.20-(-0.54)** | **0.000** | **0.000** |  | NA | . | . | . |
| **08:01:01G** | **-04:02:01G** | -0.49^a^ | -1.21.0.21 | 0.173 | . |  | NA | . | . | . |
| **08:02:01G** | **-03:01:01G** | -0.08^a^ | -0.66-0.48 | 0.760 | . |  | 1.11 | 0.54-2.25 | 0.768 | . |
| **08:02:01G** | **-04:02:01G** | -0.36^a^ | -0.70-(-0.02) | **0.037** | 4.588 |  | 0.89 | 0.51-1.54 | 0.680 | . |
| **08:02:01G** | **-05:01:01G** | 0.00^a^ | . | . | . |  | 0.34 | 0.11-1.00 | 0.051 | . |
| **08:03:02** | **-04:02:01G** | 0.00^a^ | . | . | . |  | NA | . | . | . |
| **08:04:01** | **-03:01:01G** | 0.19^a^ | -0.35-0.73 | 0.492 | . |  | 0.92 | 0.53-1.60 | 0.785 | . |
| **08:04:01** | **-03:02:01G** | 1.11 | 0.37-3.32 | 0.848 | . |  | NA | . | . | . |
| **08:04:01** | **-04:02:01G** | NA | . | . | . |  | 1.0 | . | . | . |
| **08:04:01** | **-05:01:01G** | NA | . | . | . |  | 0.92 | 0.53-1.60 | 0.785 | . |
| **08:06** | **-06:02:01G** | 0.00^a^ | . | . | . |  | NA | . | . | . |
| **08:07** | **-04:02:01G** | 0.00^a^ | . | . | . |  | 6.45 | 1.27-32.75 | **0.024** | 2.496 |
| **09:01:02G** | **-03:01:01G** | 0.01^a^ | -0.22-0.26 | 0.875 | . |  | **5.53** | **2.91-10.49** | **0.000** | **0.000** |
| **09:01:02G** | **-03:03:02G** | -0.34^a^ | -0-80-011 | 0.139 | . |  | 1.00 | . | . | . |
| **10:01:01G** | **-05:01:01G** | 0.73 | 0.09-5.66 | 0.765 | . |  | 0.85 | 0.50-1.45 | 0.565 | . |
| **11:01:01G** | **-03:01:01G** | 0.87 | 0.41-1.86 | 0.735 | . |  | 0.90 | 0.53-1.53 | 0.708 | . |
| **11:01:01G** | **-03:02:01G** | -0.09^a^ | -0.41-0.22 | 0.568 | . |  | 0.77 | 0.46-1.28 | 0.321 | . |
| **11:01:02** | **-05:01:01G** | NA | . | . | . |  | 1.00 | . | . | . |
| **11:02:01** | **-03:01:01G** | 0.08^a^ | -0.26-0.43 | 0.646 | . |  | 1.89 | 1.25-2.87 | **0.003** | 0.312 |
| **11:02:01** | **-03:02:01G** | 0.00^a^ | . | . | . |  | NA | . | . | . |
| **11:02:01** | **-04:02:01G** | NA | . | . | . |  | 1.00 | . | . | . |
| **11:02:01** | **-05:01:01G** | NA | . | . | . |  | 1.81 | 1.01-3.23 | **0.043** | . |
| **11:04:01G** | **-02:01:01G** | 2.46 | 1.07-5.67 | **0.034** | 4.216 |  | NA | . | . | . |
| **11:04:01G** | **-03:01:01G** | -0.20^a^ | -0.55-0.14 | 0.250 | . |  | 1.32 | 0.69-2.52 | 0.388 | . |
| **11:04:01G** | **-03:02:01G** | **0.65^a^** | **0.38-0.92** | **0.000** | **0.000** |  | 1.00 | . | . | . |
| **11:04:01G** | **-04:02:01G** | -0.54^a^ | -0.95-(-0.12) | **0.011** | 1.364 |  | NA | . | . | . |
| **11:04:01G** | **-05:01:01G** | **-1.27^a^** | **-1.64-(-0.90)** | **0.000** | **0.000** |  | 1.16 | 0.53-2.54 | 0.694 | . |
| **11:04:01G** | **-06:03:01G** | 0.02^a^ | -0.56-0.61 | 0.935 | . |  | NA | . | . | . |
| **11:14:01** | **-04:02:01G** | 0.25^a^ | -0.10-0.61 | 0.170 | . |  | NA | . | . | . |
| **12:01:01G** | **-03:02:01G** | **3.23^a^** | **2.73-3.73** | **0.000** | **0.000** |  | **5.53** | **2.91-10.49** | **0.000** | **0.000** |
| **13:01:01G** | **-03:01:01G** | 3.12 | 1.46-6.67 | **0.003** | 0.372 |  | 2.28 | 0.69-7.49 | 0.173 | . |
| **13:01:01G** | **-05:02:01G** | NA | . | . | . |  | 3.04 | 1.18-7.83 | **0.021** | 2.184 |
| **13:01:01G** | **-05:03:01G** | 0.00^a^ | . | . | . |  | NA | . | . | . |
| **13:01:01G** | **-06:02:01G** | **9.58** | **4.44-20.64** | **0.000** | **0.000** |  | NA | . | . | . |
| **13:01:01G** | **-06:03:01G** | 0.27^a^ | -0.17-0.72 | 0.224 | . |  | 1.05 | 0.67-1.65 | 0.818 | . |
| **13:02:01G** | **-03:01:01G** | -0.26^a^ | -0.86-0.32 | 0.377 | . |  | 0.77 | 0.18-3.34 | 0.737 | . |
| **13:02:01G** | **-05:01:01G** | 1.17 | . | . | . |  | 1.28 | 0.75-2.20 | 0.355 | . |
| **13:02:01G** | **-06:02:01G** | -0.26^a^ | -0.65-0.12 | 0.185 | . |  | 4.21 | 1.82-9.73 | **0.001** | 0.104 |
| **13:02:01G** | **-06:04:01G** | -0.27^a^ | -0.74-0.18 | 0.241 | . |  | 1.00 | . | . | . |
| **13:02:01G** | **-06:09:01G** | 0.30^a^ | -0.23-0.84 | 0.272 | . |  | 1.82 | 0.69-4.73 | 0.220 | . |
| **13:03:01G** | **-03:01:01G** | -0.10^a^ | -1.46-1.24 | 0.875 | . |  | 1.24 | 0.67-2.31 | 0.483 | . |
| **13:03:01G** | **-03:02:01G** | NA | . | . | . |  | 1.00 | . | . | . |
| **13:03:01G** | **-04:02:01G** | **-1.07^a^** | **-1.44-(-0.71)** | **0.000** | **0.000** |  | NA | . | . | . |
| **13:03:01G** | **-05:01:01G** | NA | . | . | . |  | 1.24 | 0.67-2.31 | 0.483 | . |
| **13:04** | **-03:01:01G** | 4.87 | 1.30-18.27 | **0.019** | 2.356 |  | NA | . | . | . |
| **13:04** | **-04:02:01G** | -0.74^a^ | -1.26-(-0.21) | **0.006** | 0.744 |  | NA | . | . | . |
| **13:05:01** | **-03:01:01G** | **2.50^a^** | **1.87-3.13** | **0.000** | **0.000** |  | 1.38 | 0.68-2.81 | 0.361 | . |
| **13:05:01** | **-03:02:01G** | **2.50^a^** | **1.87-3.13** | **0.000** | **0.000** |  | 0.83 | 0.55-1.27 | 0.414 | . |
| **14:01:01G** | **-05:03:01G** | -0.07^a^ | -0.57-0.42 | 0.768 | . |  | 4.97 | 1.79-13.74 | **0.002** | 0.208 |
| **14:01:01G** | **-06:03:01G** | 0.00^a^ | . | . | . |  | NA | . | . | . |
| **14:02:01G** | **-03:01:01G** | 0.22^a^ | -0.10-0.55 | 0.179 | . |  | 1.42 | 0.83-2.44 | 0.199 | . |
| **14:02:01G** | **-03:02:01G** | **0.69^a^** | **0.41-0.97** | **0.000** | **0.000** |  | 1.00 | . | . | . |
| **14:02:01G** | **-04:02:01G** | -0.57^a^ | -1.11-(-0.02) | **0.039** | 4.836 |  | 1.11 | 0.54-2.25 | 0.768 | . |
| **14:02:01G** | **-05:01:01G** | NA | . | . | . |  | 0.64 | 0.39-1.04 | 0.073 | . |
| **14:02:01G** | **-06:04:01G** | -0.70^a^ | -1.16-(-0.23) | **0.003** | 0.372 |  | NA | . | . | . |
| **14:02:01G** | **-06:09:01G** | 0.00^a^ | . | . | . |  | NA | . | . | . |
| **14:04:01** | **-03:01:01G** | 0.00^a^ | . | . | . |  | NA | . | . | . |
| **14:06:01** | **-06:03:01G** | 0.00^a^ | . | . | . |  | NA | . | . | . |
| **15:01:01G** | **-05:01:01G** | -0.08^a^ | -0.33-0.15 | 0.470 | . |  | 1.30 | 0.70-2.43 | 0.395 | . |
| **15:01:01G** | **-05:02:01G** | **-0.98^a^** | **-1.29-(-0.67)** | **0.000** | **0.000** |  | NA | . | . | . |
| **15:01:01G** | **-06:02:01G** | 1.05 | 0.39-2.82 | 0.917 | . |  | 0.97 | 0.56-1.67 | 0.927 | . |
| **15:01:01G** | **-06:03:01G** | **9.58** | **4.44-20.64** | **0.000** | **0.000** |  | NA | . | . | . |
| **15:01:01G** | **-06:04:01G** | 0.00^a^ | . | . | . |  | NA | . | . | . |
| **15:02:01G** | **-06:01:01G** | 0.39^a^ | -0.10-0.88 | 0.120 | . |  | 0.59 | 0.18-1.93 | 0.391 | . |
| **15:03:01G** | **-06:02:01G** | 0.10^a^ | -0.69-0.90 | 0.792 | . |  | NA | . | . | . |
| **16:01:01** | **-03:01:01G** | -0.23^a^ | -0.58-0.11 | 0.184 | . |  | NA | . | . | . |
| **16:01:01** | **-05:02:01G** | 2.77 | 0.83-9.27 | 0.097 | . |  | 1.23 | 0.55-2.76 | 0.608 | . |
| **16:01:01** | **-06:03:01G** | NA | . | . | . |  | 3.04 | 1.18-7.83 | **0.021** | 2.184 |
| **16:02:01G** | **-03:01:01G** | 1.18 | 0.64-2.15 | 0.586 | . |  | 0.66 | 0.35-1.24 | 0.198 | . |
| **16:02:01G** | **-03:02:01G** | -0.30^a^ | -1.01-0.40 | 0.406 | . |  | 0.43 | 0.25-0.74 | **0.003** | 0.312 |
| **16:02:01G** | **-03:03:02G** | 0.01^a^ | -0.22.0.26 | 0.875 | . |  | NA | . | . | . |
| **16:02:01G** | **-04:02:01G** | 1.33 | 0.73-2.41 | 0.345 | . |  | NA | . | . | . |
| **16:02:01G** | **-05:01:01G** | **2.73^a^** | **2.21-3.25** | **0.000** | **0.000** |  | NA | . | . | . |
| **16:02:01G** | **-05:02:01G** | -0.63^a^ | -1.10-(-0.15) | **0.010** | 1.240 |  | NA | . | . | . |
| **16:02:01G** | **-05:03:01G** | 0.00^a^ | . | . | . |  | NA | . | . | . |
| **16:02:01G** | **-06:03:01G** | 3.12 | 1.46-6.67 | **0.003** | 0.372 |  | 2.28 | 0.69-7.49 | 0.173 | . |
| **16:02:01G** | **-06:04:01G** | NA | . | . | . |  | 1.00 | . | . | . |
| **16:02:01G** | **-06:09:01G** | NA | . | . | . |  | 0.77 | 0.18-3.34 | 0.737 | . |

^a^ The Cox proportional hazards model did not fulfil the assumption of proportionality; a lognormal parametric model was constructed

^b^ The Bonferroni method was used for correcting *p*-values for all models

Analysis was adjusted for age, age at onset of sexual life, lifetime amount of sexual partners, family planning method, abortions and HPV infection

Alleles affecting an event are shown in green for greater probability (GP) or earlier occurrence (EO) and red for lower probability (LP) or later occurrence (LO)

Pc, corrected *p*-value; NC not calculated; NA, not applicable; 95%CI, 95% confidence interval; Coeff, regression coefficient; *DRB, DR beta 1; DQB1, DQ beta 1*

Values in bold indicate statistical significance based on 95%CI, *p*<0.05

**Supplementary Table S8.** HLA *DRB1-DQB1* haplotypes associated with *Ct* persistence and redetection

| **Haplotype** | | **Persistence** | | | |  | **Redetection** | | | |
| --- | --- | --- | --- | --- | --- | --- | --- | --- | --- | --- |
|  |  | **HR** | **95%CI** | ***p*** | **Pc^d^** |  | **HR** | **95%CI** | ***p*** | **Pc^d^** |
| ***DRB1*** | ***-DQB1*** |  |  |  |  |  |  |  |  |  |
| **01:01:01G** | **-03:01:01G** | **0.48^a^** | **0.31-0.66** | **0.000** | **0.000** |  | NA | . | . | . |
| **01:01:01G** | **-03:02:01G** | **4.76** | **2.03-11.14** | **0.000** | **0.000** |  | NA | . | . | . |
| **01:01:01G** | **-03:03:02G** | NA | . | . | . |  | NA | . | . | . |
| **01:01:01G** | **-04:02:01G** | NA | . | . | . |  | -0.32^a^ | -0.51-(-0.13) | **0.001** | 0.070 |
| **01:01:01G** | **-05:01:01G** | 1.54 | 0.76-3.11 | 0.228 | . |  | -0.18^a^ | -0.33-(-0.04) | **0.012** | 0.840 |
| **01:02:01G** | **-02:01:01G** | NA | . | . | . |  | 5.04^b^ | 1.10-23.06 | **0.037** | 2.590 |
| **01:02:01G** | **-03:01:01G** | 2.81 | 1.48-5.32 | **0.001** | 0.077 |  | 0.01^a^ | -0.08-0.11 | 0.792 | . |
| **01:02:01G** | **-03:02:01G** | NA | . | . | . |  | NA | . | . | . |
| **01:02:01G** | **-03:03:02G** | NA | . | . | . |  | NA | . | . | . |
| **01:02:01G** | **-04:02:01G** | NA | . | . | . |  | NA | . | . | . |
| **01:02:01G** | **-05:01:01G** | 1.32 | 0.55-3.13 | 0.525 | . |  | 5.30^b^ | 0.88-31.89 | 0.069 | . |
| **01:03:01** | **-02:01:01G** | **9.52** | **2.77-32.71** | **0.000** | **0.000** |  | **0.01^c^** | **0.00-0.05** | **0.000** | **0.000** |
| **01:03:01** | **-03:01:01G** | NA | . | . | . |  | NA | . | . | . |
| **01:03:01** | **-03:02:01G** | NA | . | . | . |  | NA | . | . | . |
| **01:03:01** | **-04:02:01G** | NA | . | . | . |  | NA | . | . | . |
| **01:03:01** | **-05:01:01G** | **9.52** | **2.77-32.71** | **0.000** | **0.000** |  | **0.01^c^** | **0.00-0.05** | **0.000** | **0.000** |
| **01:03:01** | **-05:02:01G** | NA | . | . | . |  | 0.00^a^ | . | . | . |
| **03:01:01G** | **-02:01:01G** | 0.30 | 0.06-1.46 | 0.139 | . |  | 16.88^b^ | 1.55-183.05 | **0.020** | 1.400 |
| **03:01:01G** | **-05:01:01G** | NA | . | . | . |  | 5.04^b^ | 1.10-23.06 | **0.037** | 2.590 |
| **03:02:01** | **-02:01:01G** | NA | . | . | . |  | NA | . | . | . |
| **03:02:01** | **-04:02:01G** | NA | . | . | . |  | -0.07^a^ | -0.20-0.05 | 0.237 | . |
| **03:02:02** | **-02:01:01G** | NA | . | . | . |  | NA | . | . | . |
| **03:02:02** | **-03:01:01G** | 0.91 | 0.51-1.61 | 0.753 | . |  | NA | . | . | . |
| **04:01:01G** | **-03:02:01G** | NA | . | . | . |  | NA | . | . | . |
| **04:02:01** | **-02:01:01G** | NA | . | . | . |  | NA | . | . | . |
| **04:02:01** | **-03:01:01G** | NA | . | . | . |  | NA | . | . | . |
| **04:02:01** | **-03:02:01G** | NA | . | . | . |  | NA | . | . | . |
| **04:02:01** | **-05:01:01G** | NA | . | . | . |  | NA | . | . | . |
| **04:03:01G** | **-02:01:01G** | **5.25** | **2.10-13.15** | **0.000** | **0.000** |  | NA | . | . | . |
| **04:03:01G** | **-03:01:01G** | NA | . | . | . |  | NA | . | . | . |
| **04:03:01G** | **-03:02:01G** | **6.89** | **2.82-16.83** | **0.000** | **0.000** |  | NA | . | . | . |
| **04:04:01** | **-02:01:01G** | NA | . | . | . |  | NA | . | . | . |
| **04:04:01** | **-03:01:01G** | NA | . | . | . |  | NA | . | . | . |
| **04:04:01** | **-03:02:01G** | 1.00 | . | . | . |  | 0.08^b^ | 0.00-1.18 | 0.067 | . |
| **04:04:01** | **-05:01:01G** | NA | . | . | . |  | NA | . | . | . |
| **04:05:01** | **-02:01:01G** | 1.12 | 0.72-1.75 | 0.593 | . |  | **-0.47^a^** | **-0.61-(-0.34)** | **0.000** | **0.000** |
| **04:05:01** | **-03:02:01G** | 1.05 | 0.32-3.44 | 0.930 | . |  | -0.09^a^ | -0.52-0.34 | 0.672 | . |
| **04:05:01** | **-03:01:01G** | **51.72** | **10.70-249.94** | **0.000** | **0.000** |  | -0.07^a^ | -0.21-0.07 | 0.327 | . |
| **04:05:01** | **-05:01:01G** | NA | . | . | . |  | NA | . | . | . |
| **04:05:04** | **-02:01:01G** | NA | . | . | . |  | 0.00^a^ | . | . | . |
| **04:05:04** | **-03:01:01G** | NA | . | . | . |  | NA | . | . | . |
| **04:05:04** | **-03:02:01G** | 1.00 | . | . | . |  | NA | . | . | . |
| **04:05:04** | **-04:02:01G** | 1.45 | 0.76-2.77 | 0.252 | . |  | 5.86^b^ | 0.91-37.68 | 0.062 | . |
| **04:05:04** | **-05:01:01G** | NA | . | . | . |  | NA | . | . | . |
| **04:07:01G** | **-02:01:01G** | 1.04 | 0.53-2.04 | 0.908 | . |  | 0.00^a^ | . | . | . |
| **04:07:01G** | **-03:01:01G** | 1.00 | 0.32-3.07 | 0.993 | . |  | 0.02^a^ | -0.06-0.11 | 0.591 | . |
| **04:07:01G** | **-03:02:01G** | 1.14 | 0.65-1.99 | 0.633 | . |  | 0.10^a^ | 0.03-0.17 | **0.004** | 0.280 |
| **04:07:01G** | **-03:02:03** | NA | . | . | . |  | 0.04^a^ | -0.08-0.17 | 0.532 | . |
| **04:07:01G** | **-04:02:01G** | 1.00 | . | . | . |  | 0.04^a^ | -0.01-0.11 | 0.164 | . |
| **04:07:01G** | **-05:01:01G** | 1.66 | 0.57-4.82 | 0.350 | . |  | NA | . | . | . |
| **04:08:01** | **-02:01:01G** | 0.70 | 0.34-1.44 | 0.338 | . |  | NA | . | . | . |
| **04:08:01** | **-03:01:01G** | 0.70 | 0.34-1.44 | 0.338 | . |  | NA | . | . | . |
| **04:10:01G** | **-04:02:01G** | NA | . | . | . |  | NA | . | . | . |
| **04:11:01** | **-02:01:01G** | 1.12 | 0.51-2.46 | 0.774 | . |  | NA | . | . | . |
| **04:11:01** | **-03:02:01G** | 0.66 | 0.35-1.26 | 0.213 | . |  | 0.10^b^ | 0.01-0.82 | **0.032** | 2.240 |
| **04:11:01** | **-04:02:01G** | 2.31 | 1.02-5.21 | **0.044** | 3.388 |  | 0.03^a^ | -0.04-0.10 | 0.393 | . |
| **07:01:01G** | **-02:01:01G** | 1.44 | 0.32-6.32 | 0.628 | . |  | **0.01^c^** | **0.00-0.05** | **0.000** | **0.000** |
| **07:01:01G** | **-03:01:01G** | NC | . | . | . |  | NA | . | . | . |
| **07:01:01G** | **-03:02:01G** | **5.25** | **2.10-13.15** | **0.000** | **0.000** |  | 0.00^a^ | . | . | . |
| **07:01:01G** | **-03:03:02G** | NA | . | . | . |  | 0.08^a^ | -0.03-0.20 | 0.155 | . |
| **07:01:01G** | **-04:02:01G** | 1.00 | . | . | . |  | 0.08^a^ | -0.03-0.20 | 0.155 | . |
| **07:01:01G** | **-05:01:01G** | **9.52** | **2.77-32.71** | **0.000** | **0.000** |  | **0.01^c^** | **0.00-0.05** | **0.000** | **0.000** |
| **07:11** | **-03:02:01G** | 1.12 | 0.51-2.46 | 0.774 | . |  | NA | . | . | . |
| **07:11** | **-04:02:01G** | NA | . | . | . |  | NA | . | . | . |
| **08:01:01G** | **-03:01:01G** | 1.45 | 0.44-4.79 | 0.538 | . |  | -0.17^a^ | -0.36-0.02 | 0.081 | . |
| **08:01:01G** | **-04:02:01G** | 1.45 | 0.44-4.79 | 0.538 | . |  | -0.17^a^ | -0.36-0.02 | 0.081 | . |
| **08:02:01G** | **-03:01:01G** | **31.40** | **5.96-165.42** | **0.000** | **0.000** |  | NA | . | . | . |
| **08:02:01G** | **-04:02:01G** | 0.68 | 0.29-1.62 | 0.394 | . |  | -0.00^a^ | -0.10-0.10 | 0.949 | . |
| **08:02:01G** | **-05:01:01G** | 1.00 | . | . | . |  | -0.32a | -0.51-(-0.13) | **0.001** | 0.070 |
| **08:03:02** | **-04:02:01G** | NA | . | . | . |  | 0.00^a^ | . | . | . |
| **08:04:01** | **-03:01:01G** | 2.81 | 1.48-5.32 | **0.001** | 0.077 |  | NA | . | . | . |
| **08:04:01** | **-03:02:01G** | NA | . | . | . |  | NA | . | . | . |
| **08:04:01** | **-04:02:01G** | 1.00 | . | . | . |  | NA | . | . | . |
| **08:04:01** | **-05:01:01G** | 2.81 | 1.48-5.32 | **0.001** | 0.077 |  | NA | . | . | . |
| **08:06** | **-06:02:01G** | NA | . | . | . |  | 0.00^a^ | . | . | . |
| **08:07** | **-04:02:01G** | NA | . | . | . |  | 0.03^a^ | -0.09-0.15 | 0.626 | . |
| **09:01:02G** | **-03:01:01G** | 1.74 | 0.89-3.39 | 0.101 | . |  | 0.03^a^ | -0.05-0.12 | 0.425 | . |
| **09:01:02G** | **-03:03:02G** | 1.74 | 0.89-3.39 | 0.101 | . |  | 0.00^a^ | . | . | . |
| **10:01:01G** | **-05:01:01G** | **0.36** | **0.21-0.62** | **0.000** | **0.000** |  | -0.10^a^ | -0.23-0.02 | 0.122 | . |
| **11:01:01G** | **-03:01:01G** | **21.83** | **5.62-84.75** | **0.000** | **0.000** |  | 2.42^b^ | 0.48-12.10 | 0.279 | . |
| **11:01:01G** | **-03:02:01G** | **51.72** | **10.70-249.94** | **0.000** | **0.000** |  | 0.07^a^ | -0.20-0.34 | 0.610 | . |
| **11:01:02** | **-05:01:01G** | NA | . | . | . |  | NA | . | . | . |
| **11:02:01** | **-03:01:01G** | 0.82 | 0.34-1.97 | 0.666 | . |  | 0.01^a^ | -0.08-0.11 | 0.792 | . |
| **11:02:01** | **-03:02:01G** | NA | . | . | . |  | NA | . | . | . |
| **11:02:01** | **-04:02:01G** | 1.00 | . | . | . |  | NA | . | . | . |
| **11:02:01** | **-05:01:01G** | NA | . | . | . |  | 0.01^a^ | -0.08-0.11 | 0.792 | . |
| **11:04:01G** | **-02:01:01G** | **10.34** | **2.87-37.24** | **0.000** | **0.000** |  | NA | . | . | . |
| **11:04:01G** | **-03:01:01G** | 2.10 | 0.78-5.63 | 0.137 | . |  | 3.52^b^ | 0.68-18.05 | 0.131 | . |
| **11:04:01G** | **-03:02:01G** | NA | . | . | . |  | NA | . | . | . |
| **11:04:01G** | **-04:02:01G** | NA | . | . | . |  | NA | . | . | . |
| **11:04:01G** | **-05:01:01G** | NA | . | . | . |  | NA | . | . | . |
| **11:04:01G** | **-06:03:01G** | NA | . | . | . |  | NA | . | . | . |
| **11:14:01** | **-04:02:01G** | NA | . | . | . |  | NA | . | . | . |
| **12:01:01G** | **-03:02:01G** | NA | . | . | . |  | 0.03^a^ | -0.05-0.12 | 0.425 | . |
| **13:01:01G** | **-03:01:01G** | 1.00 | . | . | . |  | -0.02^a^ | -0.17-0.12 | 0.768 | . |
| **13:01:01G** | **-05:02:01G** | NA | . | . | . |  | **-0.17^a^** | **-0.27-(-0.08)** | **0.000** | **0.000** |
| **13:01:01G** | **-05:03:01G** | 1.00 | . | . | . |  | 0.00^a^ | . | . | . |
| **13:01:01G** | **-06:02:01G** | 0.11^a^ | -0.07-0.29 | 0.235 | . |  | NA | . | . | . |
| **13:01:01G** | **-06:03:01G** | 0.52 | 0.27-1.03 | 0.064 | . |  | -0.02^a^ | -0.13-0.08 | 0.662 | . |
| **13:02:01G** | **-03:01:01G** | 0.48 | 0.15-1.52 | 0.216 | . |  | 0.10^a^ | -0.00-0.21 | 0.060 | . |
| **13:02:01G** | **-05:01:01G** | NA | . | . | . |  | NA | . | . | . |
| **13:02:01G** | **-06:02:01G** | NA | . | . | . |  | 6.06^b^ | 0.96-38.36 | 0.055 | . |
| **13:02:01G** | **-06:04:01G** | 0.39 | 0.12-1.24 | 0.111 | . |  | 0.00^a^ | . | . | . |
| **13:02:01G** | **-06:09:01G** | 1.17 | 0.50-2.76 | 0.704 | . |  | 0.10^a^ | -0.04-0.25 | 0.187 | . |
| **13:03:01G** | **-03:01:01G** | NA | . | . | . |  | -0.17^a^ | -0.36-0.02 | 0.081 | . |
| **13:03:01G** | **-03:02:01G** | NA | . | . | . |  | 0.00^a^ | . | . | . |
| **13:03:01G** | **-04:02:01G** | NA | . | . | . |  | -0.17^a^ | -0.36-0.02 | 0.081 | . |
| **13:03:01G** | **-05:01:01G** | NA | . | . | . |  | NA | . | . | . |
| **13:04** | **-03:01:01G** | 1.39 | 0.70-2.53 | 0.368 | . |  | NA | . | . | . |
| **13:04** | **-04:02:01G** | 1.45 | 0.44-4.79 | 0.538 | . |  | NA | . | . | . |
| **13:05:01** | **-03:01:01G** | **4.20** | **1.97-8.95** | **0.000** | **0.000** |  | 2.92^b^ | 1.24-6.86 | **0.014** | 0.980 |
| **13:05:01** | **-03:02:01G** | **4.20** | **1.97-8.95** | **0.000** | **0.000** |  | -0.02^a^ | -0.10-0.05 | 0.567 | . |
| **14:01:01G** | **-05:03:01G** | 3.76 | 1.76-8.03 | **0.001** | 0.077 |  | -0.02^a^ | -0.25-0.21 | 0.843 | . |
| **14:01:01G** | **-06:03:01G** | 1.00 | . | . | . |  | 0.00^a^ | . | . | . |
| **14:02:01G** | **-03:01:01G** | 0.89 | 0.42-1.88 | 0.772 | . |  | -0.04^a^ | -0.22-0.13 | 0.603 | . |
| **14:02:01G** | **-03:02:01G** | NA | . | . | . |  | NA | . | . | . |
| **14:02:01G** | **-04:02:01G** | **31.42** | **5.96-165.42** | **0.000** | **0.000** |  | NA | . | . | . |
| **14:02:01G** | **-05:01:01G** | **0.48^a^** | **0.31-0.66** | **0.000** | **0.000** |  | NA | . | . | . |
| **14:02:01G** | **-06:04:01G** | 0.48 | 0.15-1.52 | 0.216 | . |  | NA | . | . | . |
| **14:02:01G** | **-06:09:01G** | 1.00 | . | . | . |  | NA | . | . | . |
| **14:04:01** | **-03:01:01G** | NA | . | . | . |  | NA | . | . | . |
| **14:06:01** | **-06:03:01G** | 1.00 | . | . | . |  | NA | . | . | . |
| **15:01:01G** | **-05:01:01G** | 0.08^a^ | -0.12-0.30 | 0.403 | . |  | NA | . | . | . |
| **15:01:01G** | **-05:02:01G** | NA | . | . | . |  | -0.07^a^ | -0.20-0.05 | 0.237 | . |
| **15:01:01G** | **-06:02:01G** | 0.73 | 0.35-1.52 | 0.411 | . |  | 7.46^b^ | 1.58-35.20 | **0.011** | 0.770 |
| **15:01:01G** | **-06:03:01G** | 0.11^a^ | -0.07-0.29 | 0.235 | . |  | NA | . | . | . |
| **15:01:01G** | **-06:04:01G** | NA | . | . | . |  | 0.00^a^ | . | . | . |
| **15:02:01G** | **-06:01:01G** | 1.03 | 0.61-1.76 | 0.887 | . |  | NA | . | . | . |
| **15:03:01G** | **-06:02:01G** | NA | . | . | . |  | NA | . | . | . |
| **16:01:01** | **-03:01:01G** | NA | . | . | . |  | NA | . | . | . |
| **16:01:01** | **-05:02:01G** | 2.11 | 0.93-4.77 | 0.072 | . |  | **-0.17^a^** | **-0.27-(-0.08)** | **0.000** | **0.000** |
| **16:01:01** | **-06:03:01G** | NA | . | . | . |  | **-0.17^a^** | **-0.27-(-0.08)** | **0.000** | **0.000** |
| **16:02:01G** | **-03:01:01G** | 1.34 | 0.51-3.50 | 0.550 | . |  | 0.01^a^ | -0.06-0.08 | 0.798 | . |
| **16:02:01G** | **-03:02:01G** | 0.24^a^ | 0.10-0.39 | **0.001** | 0.077 |  | 0.00^a^ | -0.13-0.13 | 0.988 | . |
| **16:02:01G** | **-03:03:02G** | 1.74 | 0.89-3.39 | 0.101 | . |  | NA | . | . | . |
| **16:02:01G** | **-04:02:01G** | NA | . | . | . |  | NA | . | . | . |
| **16:02:01G** | **-05:01:01G** | NA | . | . | . |  | NA | . | . | . |
| **16:02:01G** | **-05:02:01G** | NA | . | . | . |  | NA | . | . | . |
| **16:02:01G** | **-05:03:01G** | NA | . | . | . |  | NA | . | . | . |
| **16:02:01G** | **-06:03:01G** | NA | . | . | . |  | -0.02^a^ | -0.17-0.12 | 0.768 | . |
| **16:02:01G** | **-06:04:01G** | NA | . | . | . |  | NA | . | . | . |
| **16:02:01G** | **-06:09:01G** | NA | . | . | . |  | 0.10^a^ | -0.00-0.21 | 0.060 | . |

^a^ The Cox proportional hazards model did not fulfil the assumption of proportionality; a logistic parametric model was constructed

^b^ The Cox proportional hazards model did not fulfil the assumption of proportionality; a Weibull parametric model was constructed

^c^ The Cox proportional hazards model did not fulfil the assumption of proportionality; a Gompertz parametric model was constructed

^d^ The Bonferroni method was used for correcting *p*-values for all models

Analysis was adjusted for age, age at onset of sexual life, lifetime amount of sexual partners, family planning method, abortions and HPV infection

Alleles affecting an event are shown in green for greater probability (GP) or earlier occurrence (EO) and red for lower probability (LP) or later occurrence (LO)

Pc, corrected *p*-value; NC not calculated; NA, not applicable; 95%CI, 95% confidence interval; Coeff, regression coefficient; *DRB, DR beta 1; DQB1, DQ beta 1*

Values in bold indicate statistical significance based on 95%CI, *p*<0.05

**Supplementary Table S9**. Classical and nonclassical secretion pathway predictions for *Ct* proteins

| **Protein name** | **Secretion pathway** | | |
| --- | --- | --- | --- |
|  | **Classical** | **Nonclassical** | |
|  | **SignalP 5.0** | | **SecretomeP 2.0 (score >0.5)** |
| 3-oxoacyl-[acyl-carrier protein] reductase | - | | x |
| Anti-sigma B factor antagonist RsbV | - | | x |
| ATP-dependent Clp protease proteolytic subunit ClpP | - | | - |
| ATP-dependent Clp protease proteolytic subunit ClpP-2 | - | | - |
| Heat shock protein 60 kDa family chaperone GroEL-1 | - | | - |
| Heat shock protein 60 kDa family chaperone GroEL-2 | - | | - |
| Heat shock protein 60 kDa family chaperone GroEL-3 | - | | - |
| Heat shock protein 60 kDa family chaperone GroEL-4 | - | | - |
| Hypothetical protein-CPAF | x | | - |
| Hypothetical protein-OMP-A (MOMP) | x | | - |
| Hypothetical protein-OMP-C | x | | - |
| Hypothetical protein-PMP-A | - | | x |
| Hypothetical protein-PMP-B | - | | x |
| Hypothetical protein-PMP-C | - | | x |
| Hypothetical protein-PMP-D | x | | - |
| Hypothetical protein-PMP-F | x | | - |
| Hypothetical protein-PMP-G | - | | x |
| Inclusion membrane protein-11_incA | - | | - |
| LSU ribosomal protein L6p (L9e)-Rplf | - | | x |
| NAD-dependent glyceraldehyde-3-phosphate dehydrogenase | - | | - |
| Ortholog of Bordetella pertussis | - | | - |
| Outer membrane protein B precursor | x | | - |
| Outer membrane protein H precursor-OMP-H | x | | - |
| Virulence plasmid protein (VPP) pGP3-D | - | | x |

**Supplementary Table S10**. MHC-II binding epitopes from *Ct* 3-oxoacyl-[acyl-carrier protein] reductase related to different events

| Event | Locus | Allele | Effect | Amount of peptides | Peptide | Core | %Rank |
| --- | --- | --- | --- | --- | --- | --- | --- |
| ***Infection*** | *DRB1* | *01:02 | Major probability | 1 | AIINISSIVGLRGSPGQTNY^a^ | IVGLRGSPG | 1.76 |
|  |  | *03:02 | Earlier occurrence | - | - | - | - |
|  |  | *04:10 | Later occurrence | - | - | - | - |
|  |  | *08:01 | Earlier occurrence | - | - | - | - |
|  |  | *11:01 | Later occurrence | 1 | YNVCSAVIRPMIKARSGAII | IRPMIKARS | 0.44 |
|  |  |  |  | 2 | YAAAKAGIIGFSKALSKEVG | IIGFSKALS | 1.85 |
|  |  | *12:01 |  | - | - | - | - |
|  |  | *14:02 |  | - | - | - | - |
| ***Clearance*** |  | *09:01 | Major probability | 1 | RGSPGQTNYAAAKAGIIGFS^c^ | YAAAKAGII | 0.07 |
|  |  | *12:01 |  | - | - | - | - |
| ***Persistence*** |  | *08:02 | Major probability | 1 | YNVCSAVIRPMIKARSGAII^b^ | IRPMIKARS | 0.80 |
|  |  | *10:01 | Lower probability | - | - | - | - |
|  |  | *11:01 | Major probability | 1 | YNVCSAVIRPMIKARSGAII | IRPMIKARS | 0.44 |
|  |  |  |  | 2 | YAAAKAGIIGFSKALSKEVG | IIGFSKALS | 1.85 |
|  |  | *14:01 |  | - | - | - | - |
|  |  | *14:02 |  | - | - | - | - |
|  |  | *13:05 |  | 1 | YNVCSAVIRPMIKARSGAII^a^ | IRPMIKARS | 0.44 |
|  |  |  |  | 2 | YAAAKAGIIGFSKALSKEVG^a^ | IIGFSKALS | 1.85 |
| ***Redetection*** |  | *01:03 | Earlier occurrence | 1 | GSPGQTNYAAAKAGIIGFSK^a^ | YAAAKAGII | 1.53 |
|  |  | *08:02 |  | 1 | YNVCSAVIRPMIKARSGAII^b^ | IRPMIKARS | 0.80 |
|  |  | *16:01 |  | - | - | - | - |
| ***Clearance*** | *DQB1* | *05:03 | Major probability | - | - | - | - |
| ***Persistence*** |  | *05:03 | Major probability | - | - | - | - |

^a^ Peptides binding strongly to alleles associated with effects increasing *Ct* susceptibility

^b^ Peptides binding strongly to an allele associated with two different events having effects increasing *Ct* susceptibility: GP of persistence and EO of infection

^c^ Peptides binding strongly to alleles associated with effects reducing *Ct* susceptibility

**Supplementary Table S11**. *Chlamydia* anti-sigma B factor MHC-II binding epitopes related to different events

| **Event** | **Locus** | **Allele** | **Effect** | **Amount of peptides** | **Peptide** | **Core** | **%Rank** |
| --- | --- | --- | --- | --- | --- | --- | --- |
| ***Infection*** | ***DRB1*** | *01:02 | Major probability | - | - | - | - |
|  |  | *03:02 | Earlier occurrence | - | - | - | - |
|  |  | *04:10 | Later occurrence | - | - | - | - |
|  |  | *08:01 | Earlier occurrence | 1 | GIRVLLQSYHQVGKNAGKIA | YHQVGKNAG | 1.56 |
|  |  | *11:01 | Later occurrence | 1 | IRVLLQSYHQVGKNAGKIAL | YHQVGKNAG | 0.47 |
|  |  | *12:01 |  | - | - | - | - |
|  |  | *14:02 |  | - | - | - | - |
| ***Clearance*** |  | *09:01 | Major probability | - | - | - | - |
|  |  | *12:01 |  | - | - | - | - |
| ***Persistence*** |  | *08:02 | Major probability | 1 | IRVLLQSYHQVGKNAGKIAL^b^ | YHQVGKNAG | 0.73 |
|  |  | *10:01 | Lower probability | - | - | - | - |
|  |  | *11:01 | Major probability | 1 | IRVLLQSYHQVGKNAGKIAL | YHQVGKNAG | 0.47 |
|  |  | *14:01 |  | - | - | - | - |
|  |  | *14:02 |  | - | - | - | - |
|  |  | *13:05 |  | 1 | IRVLLQSYHQVGKNAGKIAL^a^ | YHQVGKNAG | 0.47 |
| ***Redetection*** |  | *01:03 | Earlier occurrence | - | - | - | - |
|  |  | *08:02 |  | 1 | IRVLLQSYHQVGKNAGKIAL^b^ | YHQVGKNAG | 0.73 |
|  |  | *16:01 |  | - | - | - | - |
| ***Clearance*** | ***DQB1*** | *05:03 | Major probability | - | - | - | - |
| ***Persistence*** |  | *05:03 |  | - | - | - | - |

^a^ Peptides binding strongly to alleles associated with effects increasing *Ct* susceptibility

^b^ Peptides binding strongly to an allele associated with two different events having effects increasing *Ct* susceptibility: GP of persistence and EO of infection

**Supplementary Table S12**. *Chlamydia* CPAF MHC-II binding epitopes related to different events

| **Event** | **Locus** | **Allele** | **Effect** | **Amount of peptides** | **Peptide** | **Core** | **%Rank** |
| --- | --- | --- | --- | --- | --- | --- | --- |
| ***Infection*** | ***DRB1*** | *01:02 | Major probability | - | - | - | - |
|  |  | *03:02 | Earlier occurrence | - | - | - | - |
|  |  | *04:10 | Later occurrence | - | - | - | - |
|  |  | *08:01 | Earlier occurrence | - | - | - | - |
|  |  | *11:01 | Later occurrence | - | - | - | - |
|  |  |  |  | - | - | - | - |
|  |  | *12:01 |  | - | - | - | - |
|  |  | *14:02 |  | - | - | - | - |
| ***Clearance*** |  | *09:01 | Major probability | - | - | - | - |
|  |  | *12:01 |  | - | - | - | - |
| ***Persistence*** |  | *08:02 | Major probability | - | - | - | - |
|  |  | *10:01 | Lower probability | - | - | - | - |
|  |  | *11:01 | Major probability | - | - | - | - |
|  |  |  |  | - | - | - | - |
|  |  | *14:01 |  | - | - | - | - |
|  |  | *14:02 |  | - | - | - | - |
|  |  | *13:05 |  | - | - | - | - |
|  |  |  |  | - | - | - | - |
| ***Redetection*** |  | *01:03 | Earlier occurrence | - | - | - | - |
|  |  | *08:02 |  | - | - | - | - |
|  |  | *16:01 |  | - | - | - | - |
| ***Clearance*** | ***DQB1*** | *05:03 | Major probability | - | - | - | - |
| ***Persistence*** |  | *05:03 | Major probability | - | - | - | - |

**Supplementary Table S13**. *Chlamydia* OMP-A MHC-II binding epitopes related to different events

| **Event** | **Locus** | **Allele** | **Effect** | **Amount of peptides** | **Peptide** | **Core** | **%Rank** |
| --- | --- | --- | --- | --- | --- | --- | --- |
| ***Infection*** | ***DRB1*** | *01:02 | Major probability | 1 | VSFDADTIRIAQPKLAKPVL^a^ | IRIAQPKLA | 1.14 |
|  |  |  |  | 2 | AVTIETRLIDERAAHVNAQF^a^ | LIDERAAHV | 1.48 |
|  |  | *03:02 | Earlier occurrence | 1 | MRMGYYGDFVFDRVLKTDVN | FVFDRVLKT | 1.00 |
|  |  | *04:10 | Later occurrence | - | - | - | - |
|  |  | *08:01 | Earlier occurrence | - | - | - | - |
|  |  | *11:01 | Later occurrence | - | - | - | - |
|  |  | *12:01 |  | - | - | - | - |
|  |  | *14:02 |  | 1 | SGFDTANIVPNTALNQAVVE | IVPNTALNQ | 0.90 |
| ***Clearance*** |  | *09:01 | Major probability | - | - | - | - |
|  |  | *12:01 |  | - | - | - | - |
| ***Persistence*** |  | *08:02 | Major probability | - | - | - | - |
|  |  | *10:01 | Lower probability | - | - | - | - |
|  |  | *11:01 | Major probability | - | - | - | - |
|  |  | *14:01 |  | - | - | - | - |
|  |  | *14:02 |  | 1 | SGFDTANIVPNTALNQAVVE | IVPNTALNQ | 0.90 |
|  |  | *13:05 |  | - | - | - | - |
| ***Redetection*** |  | *01:03 | Earlier occurrence | 1 | VSFDADTIRIAQPKLAKPVL^a^ | IRIAQPKLA | 1.00 |
|  |  | *08:02 |  | - | - | - | - |
|  |  | *16:01 |  | - | - | - | - |
| ***Clearance*** | ***DQB1*** | *05:03 | Major probability | - | - | - | - |
| ***Persistence*** |  | *05:03 |  | - | - | - | - |

^a^ Peptides binding strongly to alleles associated with effects increasing *Ct* susceptibility

**Supplementary Table S14**. *Chlamydia* PMP-C MHC-II binding epitopes related to different events

| **Event** | **Locus** | **Allele** | **Effect** | **Amount of peptides** | **Peptide** | **Core** | **%Rank** |
| --- | --- | --- | --- | --- | --- | --- | --- |
| ***Infection*** | ***DRB1*** | *01:02 | Major probability | 1 | EDTLSPGVTVLEAAGAQISC^a^ | VTVLEAAGA | 1.63 |
|  |  | *03:02 | Earlier occurrence | - | - | - | - |
|  |  | *04:10 | Later occurrence | 1 | MAESLSTNVISLADTKAKDN^c^ | VISLADTKA | 1.93 |
|  |  | *08:01 | Earlier occurrence | - | - | - | - |
|  |  | *11:01 | Later occurrence | 1 | ITGNTVVFDSLPRLGSKETV | FDSLPRLGS | 0.38 |
|  |  |  |  | 2 | LNPGESLQYKVLVRAQTPGQ | YKVLVRAQT | 0.48 |
|  |  |  |  | 3 | GSKETVEFSVTLKAVSAGDA | FSVTLKAVS | 1.74 |
|  |  | *12:01 |  | - | - | - | - |
|  |  | *14:02 |  | 1 | CLRCPVVYKINIVNQGTATA | YKINIVNQG | 0.85 |
|  |  |  |  | 2 | YVCKPVEYVISVSNPGDLVL | YVISVSNPG | 1.53 |
| ***Clearance*** |  | *09:01 | Major probability | 1 | SYVCKPVEYVISVSNPGDLV^c^ | YVISVSNPG | 0.76 |
|  |  |  |  | 2 | VENPVPDGYAHSSGQRVLTF^c^ | YAHSSGQRV | 1.10 |
|  |  | *12:01 |  | - | - | - | - |
| ***Persistence*** |  | *08:02 | Major probability | 1 | NPGESLQYKVLVRAQTPGQF^b^ | YKVLVRAQT | 1.33 |
|  |  |  |  | 2 | CVGENTVYRICVTNRGSAED^b^ | YRICVTNRG | 1.65 |
|  |  |  |  | 3 | RCPVVYKINIVNQGTATARN^b^ | YKINIVNQG | 1.89 |
|  |  |  |  | 4 | YVCKPVEYVISVSNPGDLVL^b^ | YVISVSNPG | 1.98 |
|  |  | *10:01 | Lower probability | 1 | YVCKPVEYVISVSNPGDLVL^a^ | YVISVSNPG | 1.55 |
|  |  | *11:01 | Major probability | 1 | ITGNTVVFDSLPRLGSKETV | FDSLPRLGS | 0.38 |
|  |  |  |  | 2 | LNPGESLQYKVLVRAQTPGQ | YKVLVRAQT | 0.48 |
|  |  |  |  | 3 | GSKETVEFSVTLKAVSAGDA | FSVTLKAVS | 1.74 |
|  |  | *14:01 |  | - | - | - | - |
|  |  | *14:02 |  | 1 | CLRCPVVYKINIVNQGTATA | YKINIVNQG | 0.85 |
|  |  |  |  | 2 | YVCKPVEYVISVSNPGDLVL | YVISVSNPG | 1.53 |
|  |  | *13:05 |  | 1 | ITGNTVVFDSLPRLGSKETV^a^ | FDSLPRLGS | 0.38 |
|  |  |  |  | 2 | LNPGESLQYKVLVRAQTPGQ^a^ | YKVLVRAQT | 0.48 |
|  |  |  |  | 3 | GSKETVEFSVTLKAVSAGDA^a^ | FSVTLKAVS | 1.74 |
| ***Redetection*** |  | *01:03 | Earlier occurrence | - | - | - | - |
|  |  | *08:02 |  | 1 | NPGESLQYKVLVRAQTPGQF^b^ | YKVLVRAQT | 1.33 |
|  |  |  |  | 2 | CVGENTVYRICVTNRGSAED^b^ | YRICVTNRG | 1.65 |
|  |  |  |  | 3 | RCPVVYKINIVNQGTATARN^b^ | YKINIVNQG | 1.89 |
|  |  |  |  | 4 | YVCKPVEYVISVSNPGDLVL^b^ | YVISVSNPG | 1.98 |
|  |  | *16:01 |  | 1 | YVCKPVEYVISVSNPGDLVL^a^ | YVISVSNPG | 1.04 |
| ***Clearance*** | ***DQB1*** | *05:03 | Major probability | - | - | - | - |
| ***Persistence*** |  | *05:03 |  | - | - | - | - |

^a^ Peptides binding strongly to alleles associated with effects increasing *Ct* susceptibility

^b^ Peptides binding strongly to an allele associated with two different events having effects increasing *Ct* susceptibility: GP of persistence and EO of infection

^c^ Peptides binding strongly to alleles associated with effects reducing *Ct* susceptibility

**Supplementary Table S15**. *Chlamydia* PMP-A MHC-II binding epitopes related to different events

| **Event** | **Locus** | **Allele** | **Effect** | **Amount of peptides** | **Peptide** | **Core** | **%Rank** |
| --- | --- | --- | --- | --- | --- | --- | --- |
| ***Infection*** | ***DRB1*** | *01:02 | Major probability | - | - | - | - |
|  |  | *03:02 | Earlier occurrence | 1 | SHMFGDSFVADLPEHITSEG | FVADLPEHI | 0.56 |
|  |  |  |  | 2 | SSCPEGTNYIINRKLILSDF | YIINRKLIL | 1.17 |
|  |  | *04:10 | Later occurrence | 1 | ANKKGGAIYAQYVNLEQNQD^c^ | IYAQYVNLE | 1.40 |
|  |  | *08:01 | Earlier occurrence | - | - | - | - |
|  |  | *11:01 | Later occurrence | 1 | RKLILSDFSLLNKVSSGGAF | FSLLNKVSS | 0.84 |
|  |  |  |  | 2 | FSESSIEFTDLRKLVAFGSE | FTDLRKLVA | 0.90 |
|  |  |  |  | 3 | FRNLAGKISFLGKNSSASIH | ISFLGKNSS | 1.80 |
|  |  | *12:01 |  | 1 | SGDFAGSRILFLNNQQITFE^d^ | ILFLNNQQI | 1.49 |
|  |  |  |  | 2 | EDVANRTSIFNQPVHLYNGT^d^ | IFNQPVHLY | 1.68 |
|  |  | *14:02 |  | 1 | SCPEGTNYIINRKLILSDFS | YIINRKLIL | 1.47 |
| ***Clearance*** |  | *09:01 | Major probability | 1 | PFFSPKGMIVFSGANLLDDA^c^ | IVFSGANLL | 1.11 |
|  |  | *12:01 |  | 1 | SGDFAGSRILFLNNQQITFE^d^ | ILFLNNQQI | 1.49 |
|  |  |  |  | 2 | EDVANRTSIFNQPVHLYNGT^d^ | IFNQPVHLY | 1.68 |
| ***Persistence*** |  | *08:02 | Major probability | - | - | - | - |
|  |  | *10:01 | Lower probability | - | - | - | - |
|  |  | *11:01 | Major probability | 1 | RKLILSDFSLLNKVSSGGAF | FSLLNKVSS | 0.84 |
|  |  |  |  | 2 | FSESSIEFTDLRKLVAFGSE | FTDLRKLVA | 0.90 |
|  |  |  |  | 3 | FRNLAGKISFLGKNSSASIH | ISFLGKNSS | 1.80 |
|  |  | *14:01 |  | 1 | IAFSGNTMLHITKKASLDRH^a^ | LHITKKASL | 0.54 |
|  |  | *14:02 |  | 1 | SCPEGTNYIINRKLILSDFS | YIINRKLIL | 1.47 |
|  |  | *13:05 |  | 1 | RKLILSDFSLLNKVSSGGAF^a^ | FSLLNKVSS | 0.84 |
|  |  |  |  | 2 | FSESSIEFTDLRKLVAFGSE^a^ | FTDLRKLVA | 0.90 |
|  |  |  |  | 3 | FRNLAGKISFLGKNSSASIH^a^ | ISFLGKNSS | 1.80 |
| ***Redetection*** |  | *01:03 | Earlier occurrence | - | - | - | - |
|  |  | *08:02 |  | - | - | - | - |
|  |  | *16:01 |  | - | - | - | - |
| ***Clearance*** | ***DQB1*** | *05:03 | Major probability | - | - | - | - |
| ***Persistence*** |  | *05:03 | Major probability | - | - | - | - |

^a^ Peptides binding strongly to alleles associated with effects increasing *Ct* susceptibility

^c^ Peptides binding strongly to alleles associated with effects reducing *Ct* susceptibility

^d^ Peptides binding strongly to an allele associated with two different events having effects reducing *Ct* susceptibility: GP of clearance and LO of infection

**Supplementary Table S16**. *Chlamydia* PMP-B MHC-II binding epitopes related to different events

| **Event** | **Locus** | **Allele** | **Effect** | **Amount of peptides** | **Peptide** | **Core** | **%Rank** |
| --- | --- | --- | --- | --- | --- | --- | --- |
| ***Infection*** | ***DRB1*** | *01:02 | Major probability | 1 | EQKEGSKLIMEPGAVLSNQN^a^ | LIMEPGAVL | 1.82 |
|  |  |  |  | 2 | WVSGVGTFLAQQGTPLSEEF^a^ | FLAQQGTPL | 1.90 |
|  |  | *03:02 | Earlier occurrence | 1 | DTDLLIDYVVDTTISKNTAK^a^ | YVVDTTISK | 0.57 |
|  |  |  |  | 2 | YYSRGTSVAIDAKPRQDFIL^a^ | VAIDAKPRQ | 0.72 |
|  |  |  |  | 3 | GWLADLRISMDLKEPSKDSS^a^ | ISMDLKEPS | 1.02 |
|  |  |  |  | 4 | GVVSYGHIKHDTTTLYPSIH^a^ | IKHDTTTLY | 1.52 |
|  |  | *04:10 | Later occurrence | 1 | TCENSHRLQFLKNSSDKQGG^c^ | LQFLKNSSD | 0.65 |
|  |  |  |  | 2 | SITNITGIIEIANNKATDVG^c^ | IIEIANNKA | 1.44 |
|  |  |  |  | 3 | VSFENITSLKVQTNGAEEKG^c^ | LKVQTNGAE | 1.55 |
|  |  | *08:01 | Earlier occurrence | - | - | - | - |
|  |  | *11:01 | Later occurrence | 1 | ASVYGGKFLYFLLNKQHGWA | YFLLNKQHG | 1.67 |
|  |  | *12:01 |  | - | - | - | - |
|  |  | *14:02 |  | 1 | ADSQEVLFSINKATKDGGAI | FSINKATKD | 1.68 |
|  |  |  |  | 2 | QDLEEIRIKYNKAGTFETKK | IKYNKAGTF | 2.00 |
| ***Clearance*** |  | *09:01 | Major probability | - | - | - | - |
|  |  | *12:01 |  | - | - | - | - |
| ***Persistence*** |  | *08:02 | Major probability | - | - | - | - |
|  |  | *10:01 | Lower probability | 1 | RNNPVCKYRVLSSNEAGQVI^c^ | YRVLSSNEA | 0.88 |
|  |  |  |  | 2 | ITLENGSFIFERNQANKRGA^c^ | FIFERNQAN | 1.04 |
|  |  |  |  | 3 | YFHKGSEYSYQASVYGGKFL^c^ | YSYQASVYG | 1.30 |
|  |  | *11:01 | Major probability | 1 | ASVYGGKFLYFLLNKQHGWA | YFLLNKQHG | 1.67 |
|  |  | *14:01 |  | - | - | - | - |
|  |  | *14:02 |  | 1 | ADSQEVLFSINKATKDGGAI^e^ | FSINKATKD | 1.68 |
|  |  |  |  | 2 | QDLEEIRIKYNKAGTFETKK^e^ | IKYNKAGTF | 2.00 |
|  |  | *13:05 |  | 1 | ASVYGGKFLYFLLNKQHGWA^a^ | YFLLNKQHG | 1.67 |
| ***Redetection*** |  | *01:03 | Earlier occurrence | 1 | WVSGVGTFLAQQGTPLSEEF^a^ | FLAQQGTPL | 1.27 |
|  |  |  |  | 2 | TAKKGGGIYAKKAKMSRIDQ^a^ | IYAKKAKMS | 1.91 |
|  |  | *08:02 |  | - | - | - | - |
|  |  | *16:01 |  | 1 | RNNPVCKYRVLSSNEAGQVI^a^ | YRVLSSNEA | 0.48 |
|  |  |  |  | 2 | FAEKDVSFENITSLKVQTNG^a^ | FENITSLKV | 0.50 |
| ***Clearance*** | ***DQB1*** | *05:03 | Major probability | - | - | - | - |
| ***Persistence*** |  | *05:03 | Major probability | - | - | - | - |

^a^ Peptides binding strongly to alleles associated with effects increasing *Ct* susceptibility

^c^ Peptides binding strongly to alleles associated with effects reducing *Ct* susceptibility

^e^ Peptides having binding core mutations regarding invasive and non-invasive variants

**Supplementary Table S17**. *Chlamydia* PMP-D MHC-II binding epitopes related to different events

| **Event** | **Locus** | **Allele** | **Effect** | **Amount of peptides** | **Peptide** | **Core** | **%Rank** |
| --- | --- | --- | --- | --- | --- | --- | --- |
| ***Infection*** | ***DRB1*** | *01:02 | Major probability | 1 | FAGKESHITALNATEGHAIV^a^ | ITALNATEG | 1.52 |
|  |  |  |  | 2 | SVDFSRNIASLGGGALQASE^a^ | IASLGGGAL | 1.66 |
|  |  |  |  | 3 | RTLSAENLVAIDGYKGAYGG^a^ | LVAIDGYKG | 1.93 |
|  |  | *03:02 | Earlier occurrence | 1 | ISWNPTGYRLDPQKAGALVF | YRLDPQKAG | 1.52 |
|  |  | *04:10 | Later occurrence | 1 | GAIAAQEIVSIQNNQAGISF^b^ | IVSIQNNQA | 0.49 |
|  |  |  |  | 2 | NCELVDNGYVLFRDNRGRVY^b^ | YVLFRDNRG | 1.81 |
|  |  | *08:01 | Earlier occurrence | - | - | - | - |
|  |  | *11:01 | Later occurrence | 1 | KDNIVKTFASNGKILGGGAI | FASNGKILG | 0.56 |
|  |  |  |  | 2 | AASGKVLFVANDKKTSFIEN | FVANDKKTS | 1.60 |
|  |  | *12:01 |  | 1 | ALWEEGAVLSALKNARFAHN | LSALKNARF | 0.31 |
|  |  |  |  | 2 | AILGREVAILHNAAVVFEQN | VAILHNAAV | 1.12 |
|  |  | *14:02 |  | 1 | KDNIVKTFASNGKILGGGAI | FASNGKILG | 1.51 |
| ***Clearance*** |  | *09:01 | Major probability | - | - | - | - |
|  |  | *12:01 |  | 1 | ALWEEGAVLSALKNARFAHN | LSALKNARF | 0.31 |
|  |  |  |  | 2 | AILGREVAILHNAAVVFEQN | VAILHNAAV | 1.12 |
| ***Persistence*** |  | *08:02 |  | - | - | - | - |
|  |  | *10:01 | Lower probability | - | - | - | - |
|  |  | *11:01 | Major probability | 1 | KDNIVKTFASNGKILGGGAI | FASNGKILG | 0.56 |
|  |  |  |  | 2 | AASGKVLFVANDKKTSFIEN | FVANDKKTS | 1.60 |
|  |  | *14:01 |  | 1 | GKILGGGAILATGKVEITNN^a^ | ILATGKVEI | 0.38 |
|  |  |  |  | 2 | ANFANGGAIAASGKVLFVAN^a^ | IAASGKVLF | 1.58 |
|  |  | *14:02 |  | 1 | KDNIVKTFASNGKILGGGAI | FASNGKILG | 1.51 |
|  |  | *13:05 |  | 1 | KDNIVKTFASNGKILGGGAI^a^ | FASNGKILG | 0.56 |
|  |  |  |  | 2 | AASGKVLFVANDKKTSFIEN^a^ | FVANDKKTS | 1.60 |
| ***Redetection*** |  | *01:03 | Earlier occurrence | 1 | TQEEFPLFSKKEGRPLSSGY^a^ | FSKKEGRPL | 1.87 |
|  |  | *08:02 |  | - | - | - | - |
|  |  | *16:01 |  | 1 | CELVDNGYVLFRDNRGRVYG^a^ | YVLFRDNRG | 1.03 |
|  |  |  |  | 2 | GAIAAQEIVSIQNNQAGISF^a^ | IVSIQNNQA | 1.49 |
| ***Clearance*** | ***DQB1*** | *05:03 | Major probability | - | - | - | - |
| ***Persistence*** |  | *05:03 |  | - | - | - | - |

^a^ Peptides binding strongly to alleles associated with effects increasing *Ct* susceptibility

^b^ Peptides binding strongly to an allele associated with two different events having effects increasing *Ct* susceptibility: GP of persistence and EO of infection

**Supplementary Table S18.** *Chlamydia* PMP-F MHC-II binding epitopes related to different events

| **Event** | **Locus** | **Allele** | **Effect** | **Amount of peptides** | **Peptide** | **Core** | **%Rank** |
| --- | --- | --- | --- | --- | --- | --- | --- |
| ***Infection*** | ***DRB1*** | *01:02 | Major probability | - | - | - | - |
|  |  | *03:02 | Earlier occurrence | 1 | VNWAPVGYIVDPIRRGDLIA^a^ | YIVDPIRRG | 1.06 |
|  |  |  |  | 2 | LRNNKGSIYFDNNTATHAGG^a^ | IYFDNNTA | 1.93 |
|  |  | *04:10 | Later occurrence | 1 | RGKAEQPILSIETTNDGQLG^c^ | ILSIETTND | 0.45 |
|  |  | *08:01 | Earlier occurrence | - | - | - | - |
|  |  | *11:01 | Later occurrence | - | - | - | - |
|  |  | *12:01 |  | 1 | GAIRSGGPIRFLNNQDVLFY^d^ | IRFLNNQDV | 1.14 |
|  |  | *14:02 |  | 1 | SNNSGPIVFNYNQGGKGGAI^e^ | IVFNYNQGG | 0.96 |
|  |  |  |  | 2 | LLGVRKLFHINNNIVTPYTL^e^ | FHINNNIVT | 1.08 |
| ***Clearance*** |  | *09:01 | Major probability | 1 | LTPFAQALFSRTEPASIRES^c^ | FSRTEPASI | 1.44 |
|  |  | *12:01 |  | 1 | GAIRSGGPIRFLNNQDVLFY^d^ | IRFLNNQDV | 1.14 |
| ***Persistence*** |  | *08:02 |  | - | - | - | - |
|  |  | *10:01 | Lower probability | - | - | - | - |
|  |  | *11:01 | Major probability | - | - | - | - |
|  |  | *14:01 |  | - | - | - | - |
|  |  | *14:02 |  | 1 | SNNSGPIVFNYNQGGKGGAI^e^ | IVFNYNQGG | 0.96 |
|  |  |  |  | 2 | LLGVRKLFHINNNIVTPYTL^e^ | FHINNNIVT | 1.08 |
|  |  | *13:05 |  | - | - | - | - |
| ***Redetection*** |  | *01:03 | Earlier occurrence | - | - | - | - |
|  |  | *08:02 |  | - | - | - | - |
|  |  | *16:01 |  | - | - | - | - |
| ***Clearance*** | ***DQB1*** | *05:03 | Major probability | - | - | - | - |
| ***Persistence*** |  | *05:03 |  | - | - | - | - |

^a^ Peptides binding strongly to alleles associated with effects increasing *Ct* susceptibility

^c^ Peptides binding strongly to alleles associated with effects reducing *Ct* susceptibility

^d^ Peptides binding strongly to an allele associated with two different events having effects reducing *Ct* susceptibility: GP of clearance and LO of infection

^e^ Peptides having binding core mutations regarding invasive and non-invasive variants

**Supplementary Table S19**. *Chlamydia* PMP-G MHC-II binding epitopes related to different events

| **Event** | **Locus** | **Allele** | **Effect** | **Amount of peptides** | **Peptide** | **Core** | **%Rank** |
| --- | --- | --- | --- | --- | --- | --- | --- |
| ***Infection*** | ***DRB1*** | *01:02 | Major probability | 1 | SMGSGGKITTLRAKAGHQIL^a^ | ITTLRAKAG | 1.99 |
|  |  | *03:02 | Earlier occurrence | 1 | MANEAPIAFVANVAGVRGGG | FVANVAGVR | 1.55 |
|  |  |  |  | 2 | LSADYGDMIFDGNLKRTAKE | MIFDGNLKR | 1.65 |
|  |  | *04:10 | Later occurrence | 1 | VAGVRGGGIAAVQDGQQGVS^c^ | IAAVQDGQQ | 1.96 |
|  |  | *08:01 | Earlier occurrence | - | - | - | - |
|  |  | *11:01 | Later occurrence | 1 | CFGNLLGSFTVLGRGHSLTF | FTVLGRGHS | 0.88 |
|  |  | *12:01 |  | 1 | EIGVGLPIVITPSKLYLNEL^d^ | IVITPSKLY | 0.54 |
|  |  |  |  | 2 | GTIYSKTDLLLLNNEKFSFY^d^ | LLLLNNEKF | 1.24 |
|  |  | *14:02 |  | 1 | MANEAPIAFVANVAGVRGGG | FVANVAGVR | 1.43 |
|  |  | *09:01 | Major probability | - | - | - | - |
| ***Clearance*** |  | *12:01 |  | 1 | EIGVGLPIVITPSKLYLNEL^d^ | IVITPSKLY | 0.54 |
|  |  |  |  | 2 | GTIYSKTDLLLLNNEKFSFY^d^ | LLLLNNEKF | 1.24 |
| ***Persistence*** |  | *08:02 |  | - | - | - | - |
|  |  | *10:01 | Lower probability | - | - | - | - |
|  |  | *11:01 | Major probability | 1 | CFGNLLGSFTVLGRGHSLTF | FTVLGRGHS | 0.88 |
|  |  | *14:01 |  | - | - | - | - |
|  |  | *14:02 |  | 1 | MANEAPIAFVANVAGVRGGG | FVANVAGVR | 1.43 |
|  |  | *13:05 |  | 1 | CFGNLLGSFTVLGRGHSLTF^a^ | FTVLGRGHS | 0.88 |
| ***Redetection*** |  | *01:03 | Earlier occurrence | 1 | GSGGKITTLRAKAGHQILFN^a^ | LRAKAGHQI | 1.64 |
|  |  | *08:02 |  | - | - | - | - |
|  |  | *16:01 |  | 1 | VSFDGEGVVFFSSNVAAGKG^a^ | VVFFSSNVA | 0.72 |
|  |  |  |  | 2 | RDALGQGYRYISGGYSLGAN^a^ | YRYISGGYS | 1.59 |
| ***Clearance*** | ***DQB1*** | *05:03 | Major probability | - | - | - | - |
| ***Persistence*** |  | *05:03 |  | - | - | - | - |

^a^ Peptides binding strongly to alleles associated with effects increasing *Ct* susceptibility

^c^ Peptides binding strongly to alleles associated with effects reducing *Ct* susceptibility

^d^ Peptides binding strongly to an allele associated with two different events having effects reducing *Ct* susceptibility: GP of clearance and LO of infection

**Supplementary Table S20**. *Chlamydia* OMP-B protein MHC-II binding epitopes related to different events

| **Event** | **Locus** | **Allele** | **Effect** | **Amount of peptides** | **Peptide** | **Core** | **%Rank** |
| --- | --- | --- | --- | --- | --- | --- | --- |
| ***Infection*** | ***DRB1*** | *01:02 | Major probability | - | - | - | - |
|  |  | *03:02 | Earlier occurrence | 1 | SQANPEVFIADSDGKLNFKE | FIADSDGKL | 1.15 |
|  |  | *04:10 | Later occurrence | - | - | - | - |
|  |  | *08:01 | Earlier occurrence | - | - | - | - |
|  |  | *11:01 | Later occurrence | - | - | - | - |
|  |  | *12:01 |  | - | - | - | - |
|  |  | *14:02 |  | - | - | - | - |
| ***Clearance*** |  | *09:01 | Major probability | 1 | RHASCPIDYIIANSQANPEV^c^ | YIIANSQAN | 1.90 |
|  |  | *12:01 | Major probability | - | - | - | - |
| ***Persistence*** |  | *08:02 | Major probability | 1 | HASCPIDYIIANSQANPEVF^b^ | YIIANSQAN | 1.97 |
|  |  | *10:01 | Lower probability | 1 | HASCPIDYIIANSQANPEVF^c^ | YIIANSQAN | 0.34 |
|  |  | *11:01 | Major probability | - | - | - | - |
|  |  | *14:01 |  | - | - | - | - |
|  |  | *14:02 |  | 1 | HASCPIDYIIANSQANPEVF | YIIANSQAN | 3.79 |
|  |  | *13:05 |  | - | - | - | - |
| ***Redetection*** |  | *01:03 | Earlier occurrence | - | - | - | - |
|  |  | *08:02 |  | 1 | HASCPIDYIIANSQANPEVF^b^ | YIIANSQAN | 1.97 |
|  |  | *16:01 |  | 1 | YRLPMNAYRDFTSEPLNSES^a^ | YRDFTSEPL | 1.24 |
| ***Clearance*** | ***DQB1*** | *05:03 | Major probability | - | - | - | - |
| ***Persistence*** |  | *05:03 | Major probability | - | - | - | - |

^a^ Peptides binding strongly to alleles associated with effects increasing *Ct* susceptibility

^b^ Peptides binding strongly to an allele associated with two different events having effects increasing *Ct* susceptibility: GP of persistence and EO of infection

^c^ Peptides binding strongly to alleles associated with effects reducing *Ct* susceptibility

**Supplementary Table S21.** C*hlamydia* OMP-C MHC-II binding epitopes related to different events

| **Event** | **Locus** | **Allele** | **Effect** | **Amount of peptides** | **Peptide** | **Core** | **%Rank** |
| --- | --- | --- | --- | --- | --- | --- | --- |
|  |  | *03:02 | Later occurrence | - | - | - | - |
| ***No infection*** | ***DQB1*** | *08:01 |  | - | - | - | - |
|  |  | *13:03 |  | - | - | - | - |
| ***Infection*** |  | *01:02 | Major probability | 1 | EDTLSPGVTVLEAAGAQISC^a^ | VTVLEAAGA | 1.63 |
|  |  | *03:02 | Earlier occurrence | - | - | - | - |
|  |  | *04:10 | Later occurrence | 1 | MAESLSTNVISLADTKAKD^c^ | VISLADTKA | 1.93 |
|  |  | *08:01 | Earlier occurrence | - | - | - | - |
|  |  | *11:01 | Later occurrence | 1 | ITGNTVVFDSLPRLGSKETV | FDSLPRLGS | 0.38 |
|  |  |  |  | 2 | LNPGESLQYKVLVRAQTPGQ | YKVLVRAQT | 0.48 |
|  |  |  |  | 3 | GSKETVEFSVTLKAVSAGDA | FSVTLKAVS | 1.74 |
|  |  | *12:01 |  | - | - | - | - |
|  |  | *14:02 |  | 1 | CLRCPVVYKINIVNQGTATA | YKINIVNQG | 0.85 |
|  |  |  |  | 2 | YVCKPVEYVISVSNPGDLVL | YVISVSNPG | 1.53 |
| ***Clearance*** |  | *09:01 | Major probability | 1 | SYVCKPVEYVISVSNPGDLV^c^ | YVISVSNPG | 0.76 |
|  |  |  |  | 2 | VENPVPDGYAHSSGQRVLTF^c^ | YAHSSGQRV | 1.10 |
|  |  | *12:01 |  | - | - | - | - |
| ***Persistence*** |  | *08:02 | Major probability | 1 | NPGESLQYKVLVRAQTPGQF^b^ | YKVLVRAQT | 1.33 |
|  |  |  |  | 2 | CVGENTVYRICVTNRGSAED^b^ | YRICVTNRG | 1.65 |
|  |  |  |  | 3 | RCPVVYKINIVNQGTATARN^b^ | YKINIVNQG | 1.89 |
|  |  |  |  | 4 | YVCKPVEYVISVSNPGDLVL^b^ | YVISVSNPG | 1.98 |
|  |  | *10:01 | Lower probability | 1 | YVCKPVEYVISVSNPGDLVL^a^ | YVISVSNPG | 1.55 |
|  |  | *11:01 | Major probability | 1 | ITGNTVVFDSLPRLGSKETV | FDSLPRLGS | 0.38 |
|  |  |  |  | 2 | LNPGESLQYKVLVRAQTPGQ | YKVLVRAQT | 0.48 |
|  |  |  |  | 3 | GSKETVEFSVTLKAVSAGDA | FSVTLKAVS | 1.74 |
|  |  | *14:01 |  | - | - | - | - |
|  |  | *14:02 |  | 1 | CLRCPVVYKINIVNQGTATA | YKINIVNQG | 0.85 |
|  |  |  |  | 2 | YVCKPVEYVISVSNPGDLVL | YVISVSNPG | 1.53 |
|  |  | *13:05 |  | 1 | ITGNTVVFDSLPRLGSKETV^a^ | FDSLPRLGS | 0.38 |
|  |  |  |  | 2 | LNPGESLQYKVLVRAQTPGQ^a^ | YKVLVRAQT | 0.48 |
|  |  |  |  | 3 | GSKETVEFSVTLKAVSAGDA^a^ | FSVTLKAVS | 1.74 |
| ***Redetection*** |  | *01:03 | Earlier occurrence | - | - | - | - |
|  |  | *08:02 |  | 1 | NPGESLQYKVLVRAQTPGQF | YKVLVRAQT | 1.33 |
|  |  |  |  | 2 | CVGENTVYRICVTNRGSAED | YRICVTNRG | 1.65 |
|  |  |  |  | 3 | RCPVVYKINIVNQGTATARN | YKINIVNQG | 1.89 |
|  |  |  |  | 4 | YVCKPVEYVISVSNPGDLVL | YVISVSNPG | 1.98 |
|  |  | *16:01 |  | 1 | YVCKPVEYVISVSNPGDLVL | YVISVSNPG | 1.04 |
| ***Clearance*** |  | *05:03 | Major probability | - | - | - | - |
| ***Persistence*** |  | *05:03 |  | - | - | - | - |

^a^ Peptides binding strongly to alleles associated with effects increasing *Ct* susceptibility

^b^ Peptides binding strongly to an allele associated with two different events having effects increasing *Ct* susceptibility: GP of persistence and EO of infection

^c^ Peptides binding strongly to alleles associated with effects reducing *Ct* susceptibility

**Supplementary Table S22**. *Chlamydia* OMP-H MHC-II binding epitopes related to different events

| **Event** | **Locus** | **Allele** | **Effect** | **Amount of peptides** | **Peptide** | **Core** | **%Rank** |
| --- | --- | --- | --- | --- | --- | --- | --- |
| ***Infection*** | ***DRB1*** | *01:02 | Major probability | - | - | - | - |
|  |  | *03:02 | Earlier occurrence | - | - | - | - |
|  |  | *04:10 | Later occurrence | 1 | VLLNEDIVLSIDSSADKTDA^c^ | VLSIDSSAD | 0.76 |
|  |  |  |  | 2 | SSADKTDAVIKVLDDSFQNN^c^ | IKVLDDSFQ | 1.95 |
|  |  | *08:01 | Earlier occurrence | - | - | - | - |
|  |  | *11:01 | Later occurrence | - | - | - | - |
|  |  | *12:01 |  | - | - | - | - |
|  |  | *14:02 |  | - | - | - | - |
| ***Clearance*** |  | *09:01 | Major probability | - | - | - | - |
|  |  | *12:01 | Major probability | - | - | - | - |
| ***Persistence*** |  | *08:02 | Major probability | - | - | - | - |
|  |  | *10:01 | Lower probability | 1 | AAELRKKFEDLSAEYNTAQG^c^ | FEDLSAEYN | 0.29 |
|  |  | *11:01 | Major probability | - | - | - | - |
|  |  | *14:01 |  | - | - | - | - |
|  |  | *14:02 |  | - | - | - | - |
|  |  | *13:05 |  | - | - | - | - |
| ***Redetection*** |  | *01:03 | Earlier occurrence | - | - | - | - |
|  |  | *08:02 |  | - | - | - | - |
|  |  | *16:01 |  | - | - | - | - |
| ***Clearance*** | ***DQB1*** | *05:03 | Major probability | - | - | - | - |
| ***Persistence*** |  | *05:03 | Major probability | - | - | - | - |

^c^ Peptides binding strongly to alleles associated with effects reducing *Ct* susceptibility

**Supplementary Table S23**. *Chlamydia* LSU MHC-II binding epitopes related to different events

| **Event** | **Locus** | **Allele** | **Effect** | **Amount of peptides** | **Peptide** | **Core** | **%Rank** |
| --- | --- | --- | --- | --- | --- | --- | --- |
| ***Infection*** | ***DRB1*** | *01:02 | Major probability | - | - | - | - |
|  |  | *03:02 | Earlier occurrence | - | - | - | - |
|  |  | *04:10 | Later occurrence | - | - | - | - |
|  |  | *08:01 | Earlier occurrence | - | - | - | - |
|  |  | *11:01 | Later occurrence | - | - | - | - |
|  |  | *12:01 |  | - | - | - | - |
|  |  | *14:02 |  | - | - | - | - |
| ***Clearance*** |  | *09:01 | Major probability | 1 | IAVKGNEVFVTPAAHVVDRP^c^ | VFVTPAAHV | 1.13 |
|  |  | *12:01 |  | - | - | - | - |
| ***Persistence*** |  | *08:02 | Major probability | 1 | GIRYENEYVRRKAGKAAKTG^b^ | YVRRKAGKA | 1.58 |
|  |  | *10:01 | Lower probability | - | - | - | - |
|  |  | *11:01 | Major probability | - | - | - | - |
|  |  | *14:01 |  | 1 | VEKNTLISIKGINKQLVGEF^a^ | IKGINKQLV | 0.71 |
|  |  | *14:02 |  | - | - | - | - |
|  |  | *13:05 |  | - | - | - | - |
| ***Redetection*** |  | *01:03 | Earlier occurrence | 1 | GIRYENEYVRRKAGKAAKTG^a^ | YVRRKAGKA | 0.77 |
|  |  | *08:02 |  | 1 | GIRYENEYVRRKAGKAAKTG^b^ | YVRRKAGKA | 1.58 |
|  |  | *16:01 |  | - | - | - | - |
| ***Clearance*** | ***DQB1*** | *05:03 | Major probability | - | - | - | - |
| ***Persistence*** |  | *05:03 |  | - | - | - | - |

^a^ Peptides binding strongly to alleles associated with effects increasing *Ct* susceptibility

^b^ Peptides binding strongly to an allele associated with two different events having effects increasing *Ct* susceptibility: GP of persistence and EO of infection

^c^ Peptides binding strongly to alleles associated with effects reducing *Ct* susceptibility

**Supplementary Table S24**. *Chlamydia* virulence plasmid protein MHC-II binding epitopes related to different events

| **Event** | **Locus** | **Allele** | **Effect** | **Amount of peptides** | **Peptide** | **Core** | **%Rank** |
| --- | --- | --- | --- | --- | --- | --- | --- |
| ***Infection*** | ***DRB1*** | *01:02 | Major probability | - | - | - | - |
|  |  | *03:02 | Earlier occurrence | 1 | VQDILDKIKTDPSLGLLKAF | IKTDPSLGL | 1.54 |
|  |  | *04:10 | Later occurrence | - | - | - | - |
|  |  | *08:01 | Earlier occurrence | 1 | ASRMEGGVVLALVREGDSKP | VLALVREGD | 1.45 |
|  |  | *11:01 | Later occurrence | 1 | GGTEIGKFTVTPKSSGSMFL | FTVTPKSSG | 1.36 |
|  |  | *12:01 |  | - | - | - | - |
|  |  | *14:02 |  | - | - | - | - |
| ***Clearance*** |  | *09:01 | Major probability | 1 | EPLKDQQIILGTTSTPVAAK^c^ | ILGTTSTPV | 2.05 |
|  |  | *12:01 | Major probability | - | - | - | - |
| ***Persistence*** |  | *08:02 | Major probability | - | - | - | - |
|  |  | *10:01 | Lower probability | - | - | - | - |
|  |  | *11:01 | Major probability | 1 | GGTEIGKFTVTPKSSGSMFL | FTVTPKSSG | 1.36 |
|  |  | *14:01 |  | - | - | - | - |
|  |  | *14:02 |  | - | - | - | - |
|  |  | *13:05 |  | 1 | GGTEIGKFTVTPKSSGSMFL^a^ | FTVTPKSSG | 1.36 |
| ***Redetection*** |  | *01:03 | Earlier occurrence | - | - | - | - |
|  |  | *08:02 |  | - | - | - | - |
|  |  | *16:01 |  | - | - | - | - |
| ***Clearance*** | ***DQB1*** | *05:03 | Major probability | - | - | - | - |
| ***Persistence*** |  | *05:03 | Major probability | - | - | - | - |

^a^ Peptides binding strongly to alleles associated with effects increasing *Ct* susceptibility

^c^ Peptides binding strongly to alleles associated with effects reducing *Ct* susceptibility

**Supplementary Table S25.** *Ct* variants and project information

| **Serovar** | **Genome ID** | **Strain** | **BioProject accession** | **BioSample accession** | **GenBank accessions** |
| --- | --- | --- | --- | --- | --- |
| A | 315277.5 | A/HAR-13 | [PRJNA13885](http://www.ncbi.nlm.nih.gov/bioproject/?term=PRJNA13885) | [SAMN02603498](http://www.ncbi.nlm.nih.gov/biosample/SAMN02603498) | [CP000051.CP000052](http://www.ncbi.nlm.nih.gov/nuccore/CP000051,CP000052) |
| B | 813210 | SC98 | PRJNA244648 | SAMN02808326 | JPNC01000000 |
| C | 1431547.3 | C/TW-3 | PRJNA230246 | SAMN02641532 | CP006945.1.CP006946.1 |
| D | 813136 | SQ29 | PRJNA338746 | SAMN05870230 | CP017731 |
| E | 813.93 | E-DK-20 | PRJNA316787 | SAMN04590089 | CP015304.CP015305 |
| F | 813.94 | F-6068 | PRJNA316787 | SAMN04590091 | CP015306.CP015307 |
| H | 813137 | SQ20 | PRJNA338746 | AMN05870245 | CP017732 |
| Ia | 813140 | SQ10 | PRJNA338746 | SAMN05870240 | CP017737 |
| J | 813145 | SQ05 | PRJNA338746 | SAMN05870234 | CP017742 |
| Ja | 813146 | SQ25 | PRJNA338746 | SAMN05870235 | CP017743 |
| K | 813147 | SQ15 | PRJNA338746 | SAMN05870243 | CP017745 |
| L2 | 813316 | KANU:L2 CT036::Tn | PRJNA386688 | SAMN07125098 | NHAU01000000 |
| LGV | 813132 | cdu1::Tn | PRJNA362456 | SAMN06239528 | CP019385 |

**References**

1 Paez-Gutierrez, I. A., Hernandez-Mejia, D. G., Vanegas, D., Camacho-Rodriguez, B. & Perdomo-Arciniegas, A. M. HLA-A, -B, -C, -DRB1 and -DQB1 allele and haplotype frequencies of 1463 umbilical cord blood units typed in high resolution from Bogota, Colombia. *Hum Immunol* **80**, 425-426 (2019).

2 Hurley, C. K. *et al.* Common, intermediate and well-documented HLA alleles in world populations: CIWD version 3.0.0. *HLA* **95**, 516-531 (2020).
